# Supplementary material for: Single-cell full-length total RNA sequencing uncovers dynamics of recursive splicing and enhancer RNAs
Source: Nat Commun. 2018 Feb 12;9:619. doi: 10.1038/s41467-018-02866-0 (PMC5809388; doi:10.1038/s41467-018-02866-0)
Supplement: Supplementary file 1 — Supplementary Information [file 41467_2018_2866_MOESM1_ESM.pdf]

Supplementary Note 1. The principle of RT with random displacement amplification (RT-RamDA)

RT-RamDA is a reaction for amplifying cDNA using strand displacement amplification (SDA) during reverse transcription directly from RNA as a template. This reaction consists of three essential components. The first is RNase H minus reverse transcriptase (RTase), which synthesizes cDNA and contributes to the amplification of cDNA with own strand displacement activity. RTase with RNase H activity is inappropriate for RT-RamDA because it degrades the template RNA during reverse transcription. The second is DNase I, which randomly inserts a nick into the cDNA strand in the RNA:cDNA hybrid. This nicking site becomes an origin of SDA. The third is T4 gene 32 protein (gp32), which is known as a single-strand DNA-binding protein. This protein promotes SDA and protects amplified cDNA from the nuclease activity of DNase I. The details of the reaction are as follows (please refer to Fig. 1a);

1. RT primers anneal to a RNA template. To capture non-poly(A) RNA for cDNA synthesis, oligo-dT primers and not-so-random primers (NSRs) are used in RT-RamDA. NSRs are semi-random primers designed to prevent annealing to rRNA[1,2]. 2. From the annealing site of the primer, reverse transcriptase synthesizes cDNA and creates a hybrid chain with the template RNA. 3. DNase I randomly inserts a nick only on the cDNA strand of the cDNA-RNA hybrid. 4. RTase resynthesizes cDNA from the nicking site and peels off the cDNA strand on the 3' side of the synthesis direction using strand displacement activity. Simultaneously, gp32 destabilizes the RNA:cDNA hybrid and promotes the strand displacement reaction. Subsequently, by binding to the peeled amplified cDNA, gp32 protects the cDNA from nuclease activity of DNase I. Consequently, RT-RamDA allows 30-fold higher cDNA amplification than does conventional RT (Fig. 1b).

Supplementary Note 2. Comparison of experimental workflow of single-cell RNA-seq methods

Method sensitivity is the product of the efficiency of each step for workflow of single-cell RNA-seq methods (Supplementary Fig. 1).

<u>Reverse transcription (RT)</u>: In this step, RNA converts to complementary DNA by reverse transcriptase. Since a high-throughput sequencer can only sequence DNA molecules, RNA molecules that were not reverse transcribed are not sequenced no

matter how much the amplification method in the downstream of the workflow is improved. Therefore, this step is very important in scRNA-seq, as single cells contain only trace amounts of RNA. In the existing methods, Quartz-Seq[3], SMART-Seq v4 and SUPeR-seq[4], it is necessary to add an adapter sequence (yellow box in Supplementary Fig. 1) for amplification to the RT primer; the adapter sequence often induces the low efficiency of RT. In particular, although SUPeR-seq realizes random priming by using the specialized hybrid primer in which a random primer and oligo-dT and an adapter for amplification are linked to each other, in practice, random priming hardly occurred (Fig. 2d, e). For this reason, the transcript coverage showed 3' end bias (Fig. 2a). However, RamDA-seq does not require such an adaptor sequence in RT primers, and priming is not disturbed. For this reason, RamDA-seq can capture not only poly(A) but also non-poly(A) RNAs and can cover full-length sequences of extremely long RNAs with high efficiency. Furthermore, since cDNA is amplified at the step of RT, RamDA-seq gains robustness against loss in the downstream step. Finally, because reverse transcriptase also has DNA-dependent DNA polymerase activity, even a small amount, removing genomic DNA is important when random priming is used for RT. In principle, this is not secured by SUPeR-seq.

Second-strand DNA synthesis: In this step, depending on the efficiency of conversion from single-stranded cDNA to double-stranded cDNA, of template switching and of poly(A) tailing, some molecules are dropped out. For example, in the extremely long RNAs, template switching does not occur when reverse transcriptase does not reach the 5 'end of RNA; these molecules are then lost (Fig. 2).

Whole transcriptome amplification (WTA): In this step, double-stranded cDNA is amplified to an amount usable in the sequencing library preparation step. In fact, in order to make quality control for cDNA possible, more than the necessary amount of DNA is amplified in the DNA preparation step. PCR amplification generates amplification bias depending on the length and GC% of cDNA. The fact that RamDA-seq does not require this step provides great advantage in accuracy, sensitivity and handling. RamDA-seq is also beneficial in that there is no QC after WTA.

Sequencing library preparation: In this common step, a sequence adaptor is added, and amplified cDNA is converted to sequencing-available double-stranded DNA with fragmentation. In the PCR of this step, since double-stranded cDNA is already

short and uniform in length, the amplification bias depending on length is relatively small.

Supplementary Note 3. Cell lysis conditions are critical for genomic DNA digestion and the detection of nuclear RNAs

RamDA-seq employs random priming to detect non-poly(A) RNAs enriched in the nucleus with minimal amplification of cDNA before library DNA construction. Thus, we examined optimal cell lysis condition for genomic DNA digestion and the detection of nuclear RNAs using qPCR (Supplementary Fig. 2). In general, nonionic detergent is often used for the cell lysis buffer in scRNA-seq[3-8]. However, qPCR analysis of the absolute quantification of genomic DNA clearly showed that cell lysis conditions using only NP40, a nonionic detergent, were not sufficient to expose genomic DNA in G2/M cells (Supplementary Fig. 2b). Moreover, insufficient exposure of genomic DNA resulted in insufficient digestion (Supplementary Fig. 2c). Next, we observed that the optimal concentration of Roche cell lysis buffer containing was essential for the digestion of genomic DNA and detection of nuclear RNAs (Supplementary Fig. 2c, d). These results indicate that the lysis and digestion of genomic DNA are critical for RamDA-seq to prevent contamination by library DNA derived from genomic DNA and the release of nuclear RNAs from complexes of genomic DNA and RNA.

Supplementary Note 4. Reproducibility and sensitivity of RamDA-seq using ERCC RNA spikes

To assess accuracy of RamDA-seq, we quantified the TPM of Spike-In Mix I in samples from 10 pg of total RNA using sailfish (version 0.9.2) (Supplementary Fig. 6). The maximum number of input molecules of an ERCC RNA spike was adjusted for 3,613 copies (ERCC-00130). The results clearly showed that RamDA-seq was highly reproducible among the replicates (Pearson correlation coefficient (PCC): 0.971 and Spearman correlation coefficient (SCC): 0.871), even though the ratio of Spike-In reads to total reads (0.12%) was 1/10 as compared with SMART-Seq v4 (1.46%). The correlation of TPM with the number of input molecules of ERCC RNA spikes was also high in RamDA-seq (PCC: 0.936 and SCC: 0.906).

Supplementary Note 5. RamDA-seq accurately detects non-poly(A) RNA dynamics throughout the cell cycle

To test whether RamDA-seq could be used to measure the expression profiles of non-poly(A) RNAs in biological samples, we applied it to cells collected during different cell-cycle phases. In our investigation of cell-cycle phases, we first collected mESCs in the G1, S, and G2/M phases using a cell sorter and performed RamDA-seq with these cells (Supplementary Fig. 14a,b). After quality checking the sequenced data (Supplementary Fig. 15a-d) and removing low-quality cells (Supplementary Fig. 15f), we performed diffusion map analysis. The cells were largely separated according to the derived cell-cycle phase (Supplementary Fig. 11a). This result indicates that RamDA-seq could detect variability among different cell-cycle phases within a single cell type.

Based on this observation, we hypothesized that the RamDA-seq data could be used to reconstruct a 'subjective time' for cells along the cell cycle. To address this possibility with use of information from a cell sorter, we first selected transcripts showing high variance among G1, S, and G2M, and performed diffusion map analysis. This procedure likely reconstructed a subjective time in each cell; the cells formed different clusters according to their derived cell, while the cells in each phase showed variance (Supplementary Fig. 11b). We then used DC1 as a proxy of subjective time for each cell along the cell cycle and searched for transcripts oscillating along DC1 by fitting a sine function (false discovery rate (FDR) < 0.01; see Methods). This procedure yielded 6,736 oscillating transcripts, including 567 non-poly(A) transcripts (Supplementary Fig. 11c). We confirmed that the cell-cycle markers for the G1 (*Ccnd3*), S (*Gmnn*), and G2/M (*Ccnb1*) phases were oscillating transcripts, whereas housekeeping genes such as *Actb* were not (Supplementary Fig. 11d).

Among the non-poly(A) RNAs oscillating during the cell cycle, the histone genes *Hist1h1a*, expressed in ESCs[9], and *Hist1h2ab* displayed oscillation (Supplementary Fig. 11e). The expression of these histone genes increased during the G1 phase and peaked at the G1-to-S transition (Supplementary Fig. 11e), which is consistent with previous studies reporting that the transcription of histone genes increases as cells progress from the G1 phase to the S phase[10]. This pattern was confirmed by single-cell preamplification RT-qPCR (Supplementary Fig. 11f).

This is, to our knowledge, the first report to profile the oscillation of several hundreds of non-poly(A) RNAs throughout cell cycle in single cells.

Supplementary Note 6. Detection of differentially expressed non-poly(A) transcripts using RamDA-seq

Using differentiation time-series data of RamDA-seq, we first evaluated the ability of RamDA-seq to detect three sets of non-poly(A) transcripts defined using bulk RNA-seq data: ES-enriched, PrE-enriched, and 'unchanged' between ES and PrE (see Methods). The detection rate for ES-enriched non-poly(A) RNAs was highest at 0 h and decreased over time (Supplementary Fig. 13d, left panel), while the detection rate for PrE-enriched non-poly(A) RNAs was lowest at 0 h and increased as differentiation progressed (Supplementary Fig. 13d, right panel). The detection rate for 'unchanged' non-poly(A) RNAs remained constant over time (Supplementary Fig. 13d, middle panel). Furthermore, the expression patterns detected by RamDA-seq using cells at 0, 12, and 72 h were confirmed by single-cell preamplification RT-qPCR (Supplementary Fig. 15g). These results show RamDA-seq can quantitatively detect non-poly(A) transcripts in single cells.

Supplementary Note 7. Validation of pseudotime analysis and clustering results of differentiation time-series data

We first assessed the expression patterns of the ES and PrE marker genes over pseudotime. Early-response ES marker genes (*Nanog*, *Sox2*, *Lefty1*, and *Zfp42*) were included in cluster 2, whereas a late-response ES marker gene (*Pou5f1*) was included in cluster 6 (Fig. 3c), which indicates that our analysis detected differences in response timing among ES marker genes. Moreover, the expression levels of *Sox2* (cluster 2) decreased over 12 h as pseudotime progressed, which suggests that the pseudotime analysis resolved heterogeneity in differentiation progression even among the cells collected at in the same time point. Likewise, PrE marker genes (*Sox17*, *Dab2*, and *Gata4*) were included in cluster 4, and *Gata4* expression increased as pseudotime progressed (Fig. 3c). These results show that the pseudotime and clustering analyses of RamDA-seq data clearly revealed expression changes during differentiation process.

Supplementary Note 8. Predicting potential functions of non-poly(A) RNAs by guilt-by-association analysis

Reasoning that transcripts with similar expression patterns should share biological functions, we attempted to infer potential functions of these dynamically regulated non-poly(A) transcripts by performing a functional enrichment analysis of each cluster (Supplementary Data 1). For example, in cluster 1 (late up-regulation) (Fig. 3c), genes associated with extracellular matrix (ECM) organization and collagen formation (e.g., *Itgb1* and *Col4a2*) were overrepresented. *Itgb1* encodes Beta 1 integrin, which binds to the ECM and is required for the PrE to form an epithelial layer covering under the inner cell mass[11], and *Col4a2* encodes collagen, which consists of the ECM components. Considering that cluster 1 was up-regulated after cluster 4, which includes *Sox17*, and that SOX17 regulates ECM genes[12], non-poly(A) transcripts in cluster 1 could be downstream of the gene regulatory network and specifically associated with PrE cell functions. We also found that cluster 2 (early down-regulation) genes (Fig. 3c), which included *Sox2* and *Nanog*, were enriched for terms associated with transcriptional regulation as well as regulation of signaling by "NODAL". As the expression of the transcripts in cluster 2 rapidly decreased after induction of differentiation from ESC to PrE, non-poly(A) transcripts in cluster 2 might be associated with self-renewal and the maintenance of pluripotency. Future studies of these non-poly(A) RNAs would enhance our understanding of ESC differentiation.

Supplementary Note 9. Detection of enhancer RNAs using scRNA-seq with 10 pg of RNA data

To investigate whether RamDA-seq could detect eRNAs, we used two definitions of eRNAs and compared the performance of scRNA-seq methods in detecting eRNAs using the 10 pg of RNA data.

First, we compiled lists of eRNA loci that were active (ES-active; positive control) and inactive (ES-inactive; negative control) in mESCs using the transcribed enhancer annotation identified using CAGE by the FANTOM5 project[13] (see Methods). Among the bulk RNA-seq methods, rdRNA-seq detected the largest number of ES-active eRNAs, whereas paRNA-seq detected fewer ES-active eRNAs, which is consistent with a previous report[14]. Among the scRNA-seq methods, RamDA-seq detected the transcription of the largest number (>250 on average) of ES-active loci,

while no transcription was detected at most of the ES-inactive eRNA loci (Supplementary Fig. 18a). paRNA-seq and Quartz-Seq detected transcription at fewer ES-active loci than did rdRNA-seq and RamDA-seq, respectively, which indicates that the ability of RamDA-seq to detect genes with low expression levels and/or non-poly(A) RNAs leads to the detection of more transcribed enhancers. Indeed, 80% of ES-active loci detected by RamDA-seq were non-poly(A) eRNAs (defined using un-downsampled bulk RNA-seq data; rdRNA-seq > paRNA-seq). We then used the second definition of eRNAs: non-poly(A) RNAs with enhancer-like histone modifications (see Methods). RamDA-seq and C1-RamDA-seq detected the largest number of eRNAs among scRNA-seq methods (Supplementary Fig. 18d,e). Furthermore, we aggregated the read coverage around ES-active eRNA loci. We observed that RamDA-seq methods, similarly to rdRNA-seq, showed bimodal peaks approximately 200–400 bp from the eRNA loci (Supplementary Fig. 18b,c), which indicates that RamDA-seq methods actually detected the transcription of eRNAs. Together, these results demonstrate a greater sensitivity and specificity of RamDA-seq for detecting eRNAs in single cells than other scRNA-seq methods.

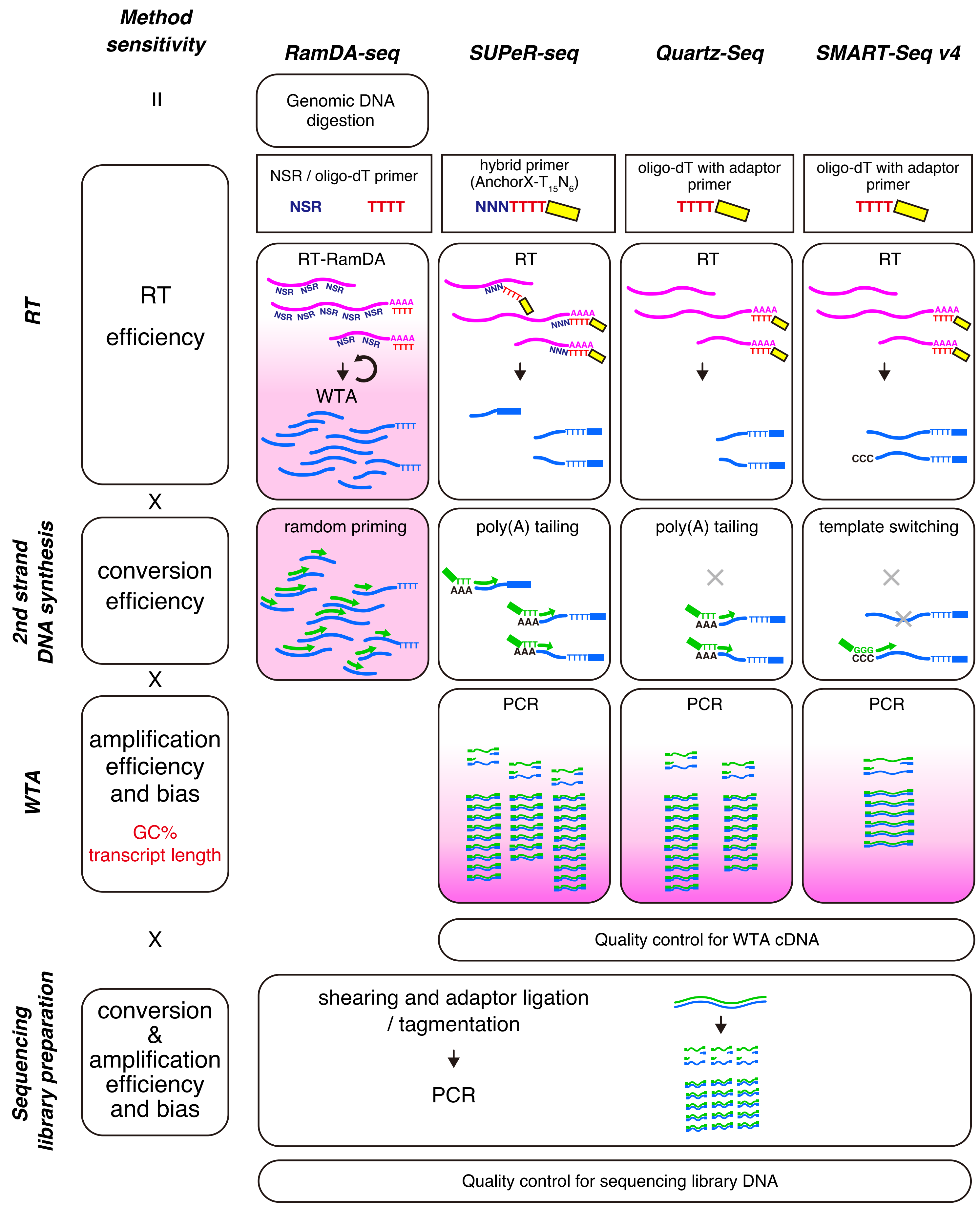

Method sensitivity
RamDA-seq
SUPeR-seq
Quartz-Seq
SMART-Seq v4
II
Genomic DNA digestion
NSR / oligo-dT primer
NSR
TTTT
hybrid primer (AnchorX-T15N6)
NNNTTTT
oligo-dT with adaptor primer
TTTT
oligo-dT with adaptor primer
TTTT
RT
RT efficiency
RT-RamDA
WTA
RT
RT
RT
CCC
X
2nd strand DNA synthesis
conversion efficiency
ramdom priming
poly(A) tailing
AAA
poly(A) tailing
AAA
template switching
GGG
CCC
X
WTA
amplification efficiency and bias
GC%
transcript length
PCR
PCR
PCR
X
Quality control for WTA cDNA
Sequencing library preparation
conversion & amplification efficiency and bias
shearing and adaptor ligation / tagmentation
PCR
Quality control for sequencing library DNA

Supplementary Figure 1. Comparison of experimental workflow of single-cell RNA-seq methods

Method sensitivity is the product of the efficiency of each step for workflow of single-cell RNA-seq methods. See Supplementary Note 2 'Comparison of experimental workflow of single-cell RNA-seq methods' for details. The magenta gradient represents cDNA amplification.

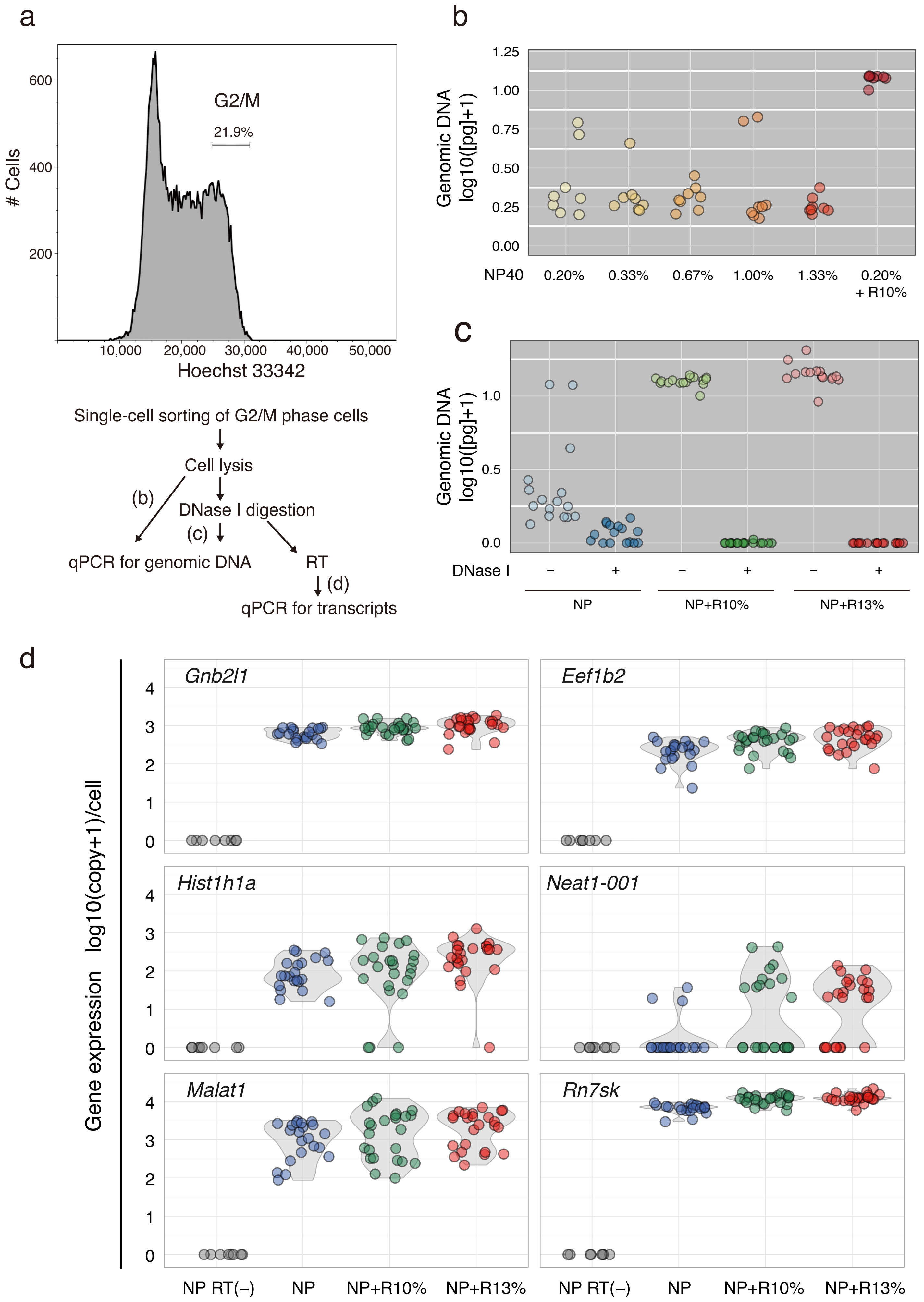
a
600
400
200
Cells
G2/M
21.9%
10,000
20,000
30,000
40,000
50,000
Hoechst 33342
Single-cell sorting of G2/M phase cells
Cell lysis
(b)
DNase I digestion
(c)
qPCR for genomic DNA
RT
(d)
qPCR for transcripts
b
Genomic DNA
log10([pg]+1)
1.25
1.00
0.75
0.50
0.25
0.00
NP40
0.20%
0.33%
0.67%
1.00%
1.33%
0.20%
+ R10%
c
Genomic DNA
log10([pg]+1)
1.0
0.5
0.0
DNase I
–
+
NP
NP+R10%
NP+R13%
d
Gene expression log10(copy+1)/cell
Gnb2l1
Eef1b2
Hist1h1a
Neat1-001
Malat1
Rn7sk
4
3
2
1
0
NP RT(–)
NP
NP+R10%
NP+R13%

Supplementary Figure 2. Cell lysis conditions are important for the decontamination of genomic DNA and detecting RNAs in the nucleus

(**a**) Single-cell sorting profile of G2/M-phase cells and scheme of the conditioning assay of the cell lysis buffer. G2/M-phase single cells were sorted depending on the intensity of Hoechst 33342, a DNA-staining fluorescent dye, using MoFlo Astrios (Beckman Coulter). (**b,c**) Genomic DNA was detected by qPCR via the detection of *Gapdh*, which is a multilocus gene; a qPCR primer that is able to detect at least 33 sites of mouse genomic DNA (analyzed by UCSC In-Silico PCR; https://genome.ucsc.edu/) was used. G2/M-phase cells had 12 pg of genomic DNA per cell, Log10(12 [pg] + 1) = 1.11. (**b**) Detected amount of genomic DNA under the different cell lysis conditions: 0.20-1.33% NP40 and 0.2% NP40 with 10% Roche cell lysis buffer, which contains NP40 and guanidinium thiocyanate. n=8. (**c**) Digestion efficiency of genomic DNA by DNase I under the different cell lysis conditions. NP: 0.3% NP40, NP+R10%: 0.3% NP40 with 10% Roche cell lysis buffer, and NP+R13%: 0.3% NP40 with 13% Roche cell lysis buffer. n=16. (**d**) Detecting nuclear RNAs and protein-coding RNAs under the different cell lysis buffer conditions using single-cell RT-qPCR. *Gnb2l1* and *Eef1b2* were typical poly(A) type protein-coding genes. *Hist1h1a* was non-poly(A) type coding genes. *Neat1-001*, *Malat1*, and *Rn7sk* were non-poly(A) type nuclear RNA. NP RT(-): 0.3% NP40 and reverse transcription negative (n=8), NP: 0.3% NP40 (n=21), NP+R10%: 0.3% NP40 with 10% Roche cell lysis buffer (n=24), and NP+R13%: 0.3% NP40 with 13% Roche cell lysis buffer (n=24).

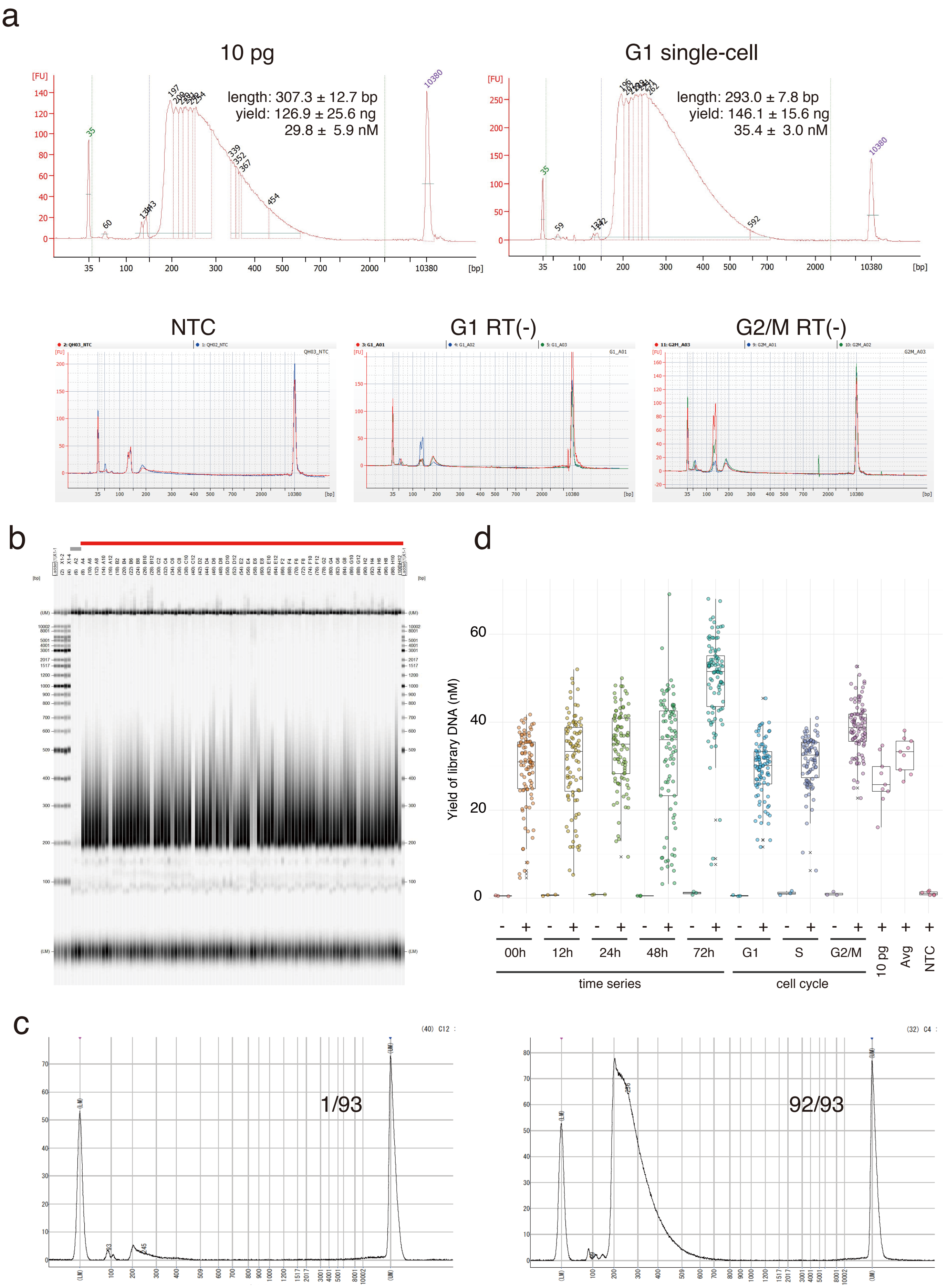
a
10 pg
length: 307.3 ± 12.7 bp
yield: 126.9 ± 25.6 ng
29.8 ± 5.9 nM
G1 single-cell
length: 293.0 ± 7.8 bp
yield: 146.1 ± 15.6 ng
35.4 ± 3.0 nM
[FU]
[bp]
NTC
G1 RT(-)
G2/M RT(-)
b
d
Yield of library DNA (nM)
00h
12h
24h
48h
72h
G1
S
G2/M
10 pg
Avg
NTC
time series
cell cycle
c
1/93
92/93

Supplementary Figure 3. Quality control of sequence library RamDA-seq DNAs

(**a**) The distribution of library DNA determined using a Bioanalyzer. The upper panels show the library DNA pattern of 10 pg of mESC total RNA and G1 phase single cells. The length and yield are displayed as averaged values from 150 to 3,000 bp (10 pg: n=6, G1 single cell: n=3). The lower panels show the overlay image of electropherograms in non-template control (NTC: n=2) and RTase minus control (RT(**-**): n=3). (**b**) Distribution of the library DNA in the three RT-minus samples (gray bar) and 93 mouse ES (0 h) single cells (red bar) analyzed by MultiNA (Shimadzu). (**c**) Instances of failure (left) and satisfactory (right) samples in b. (**d**) Yield of library DNA indicating the concentration (nM) in 24 μl of elution buffer. For sequencing, library DNAs from single cells were mixed using an equal number of molecules for each plate. (-) indicates the RT-minus control. The center line, lower and upper bounds of each box represent the median, first and third quartiles, respectively. The lower (upper) whisker extends to smallest (largest) values no further than 1.5 * inter-quartile range (IQR) from the first (third) quartile.

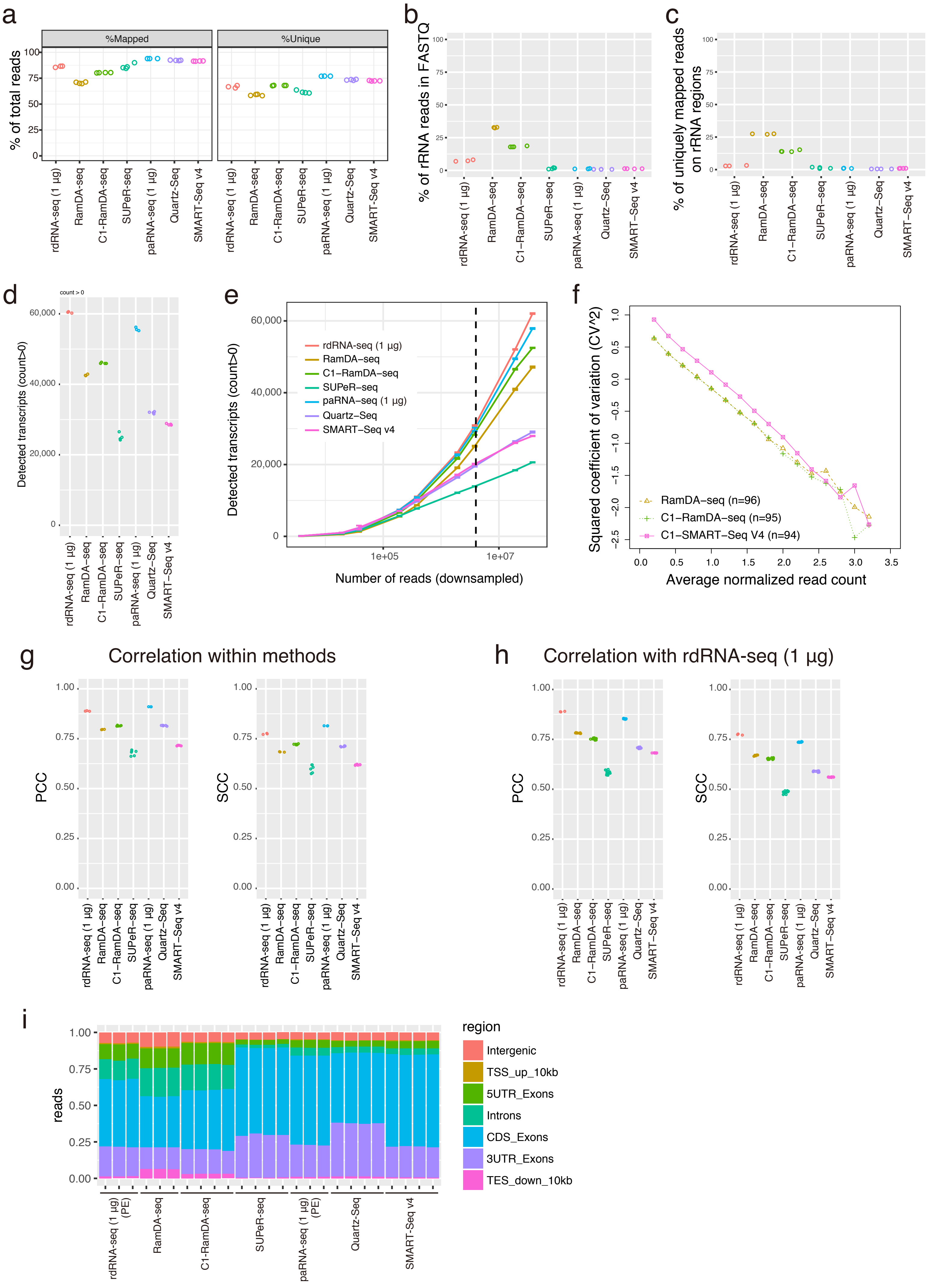
a
%Mapped
%Unique
% of total reads
rdRNA-seq (1 μg)
RamDA-seq
C1-RamDA-seq
SUPeR-seq
paRNA-seq (1 μg)
Quartz-Seq
SMART-Seq v4
b
% of rRNA reads in FASTQ
c
% of uniquely mapped reads on rRNA regions
d
count > 0
Detected transcripts (count>0)
e
Detected transcripts (count>0)
Number of reads (downsampled)
f
Squared coefficient of variation (CV^2)
Average normalized read count
RamDA-seq (n=96)
C1-RamDA-seq (n=95)
C1-SMART-Seq V4 (n=94)
g
Correlation within methods
PCC
SCC
h
Correlation with rdRNA-seq (1 μg)
i
reads
region
Intergenic
TSS_up_10kb
5UTR_Exons
Introns
CDS_Exons
3UTR_Exons
TES_down_10kb
rdRNA-seq (1 μg) (PE)
paRNA-seq (1 μg) (PE)

Supplementary Figure 4. Quality control for the sequencing results comparing different scRNA-seq protocols

(**a**) The percentage of reads mapped to the mouse genome. The left panel includes multi-mapped reads. (**b**) The percentage of rRNA reads in FASTQ files. (**c**) The percentage of uniquely mapped reads that were overlapped with rRNA gene annotations on genome. (**d**) The number of detected transcripts (count > 0). (**e**) Dependency of the number of detected transcripts within two-fold expression changes against rdRNA-seq (count > 0) on the number of sequenced reads. The dashed line represents 4 M reads per cell. (**f**) Squared coefficient of variation of the read count for 10-pg RNA samples of RamDA-seq (n=96), C1-RamDA-seq (n=95), and C1-SMART-Seq v4 (n=94). For each sample, 1.1 M reads were subsampled. (**g**) Pearson and Spearman correlation coefficients of transcript expression levels within each method. The log10(count+1) values of all transcripts were used. (**h**) Pearson and Spearman correlation coefficients of transcript expression levels between rdRNA-seq and each method. The log10(count+1) values of all transcripts were used. (**i**) Classification and distribution of uniquely mapped reads over genome features. PE: Data from paired-end reads.

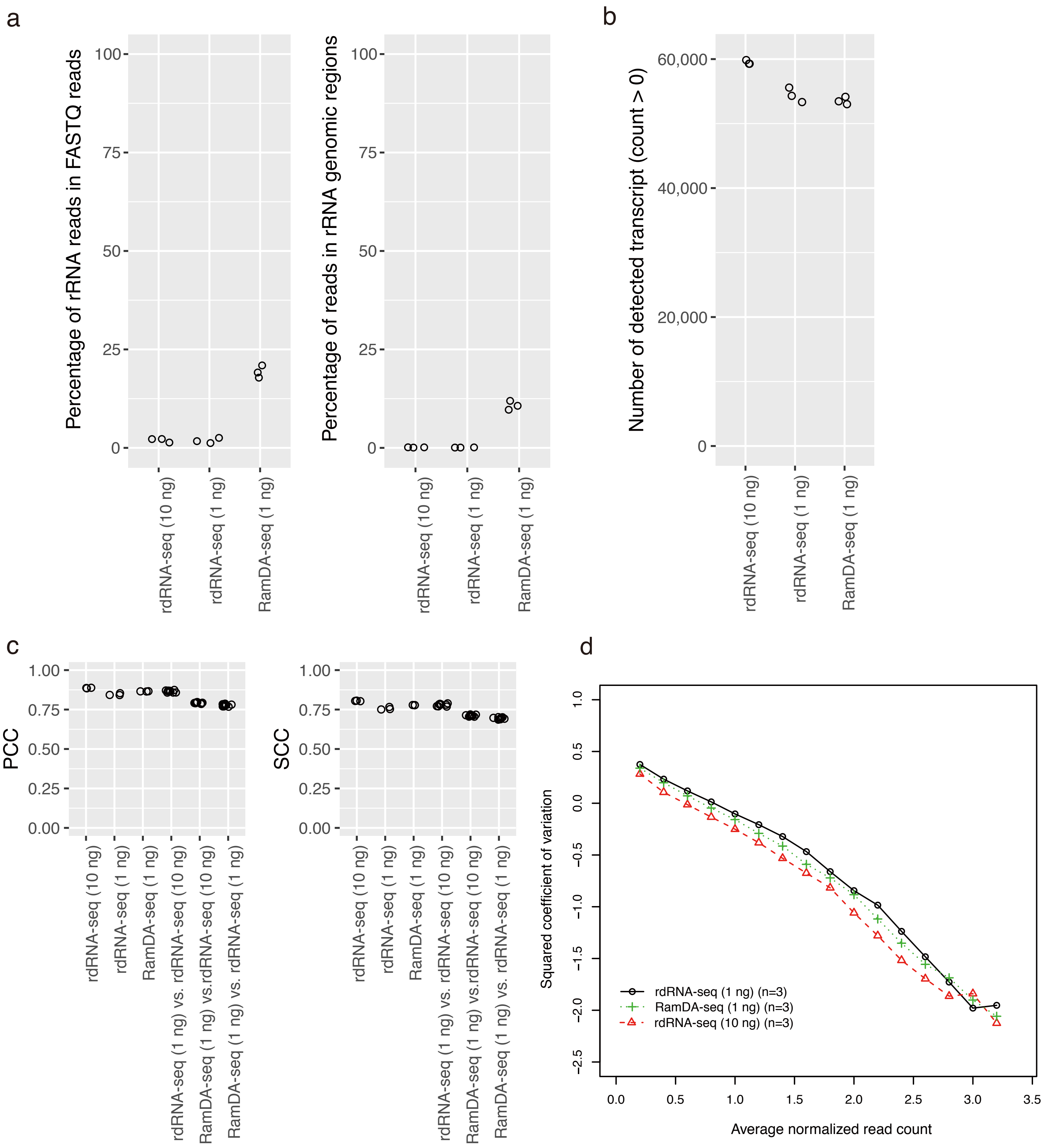

a
Percentage of rRNA reads in FASTQ reads
Percentage of reads in rRNA genomic regions
100
75
50
25
0
rdRNA-seq (10 ng)
rdRNA-seq (1 ng)
RamDA-seq (1 ng)
b
Number of detected transcript (count > 0)
60,000
40,000
20,000
0
c
PCC
SCC
1.00
0.75
0.50
0.25
0.00
rdRNA-seq (1 ng) vs. rdRNA-seq (10 ng)
RamDA-seq (1 ng) vs.rdRNA-seq (10 ng)
RamDA-seq (1 ng) vs. rdRNA-seq (1 ng)
d
Squared coefficient of variation
Average normalized read count
rdRNA-seq (1 ng) (n=3)
RamDA-seq (1 ng) (n=3)
rdRNA-seq (10 ng) (n=3)

Supplementary Figure 5. Quality control for the sequencing results of large-volume-inputted RamDA-seq using 1 ng total RNA

(**a**) The percentage of reads mapped to the rRNA sequence (28S, 18S, 16S, and 12S rRNA; left) and rRNA genomic regions (RepeatMasker; right). (**b**) The number of detected transcripts (count > 0). (**c**) Pearson (PCC) and Spearman (SCC) correlation coefficients of transcript expression levels. The log10(count+1) values of all transcripts were used. (**d**) Squared coefficient of variation of the read count.

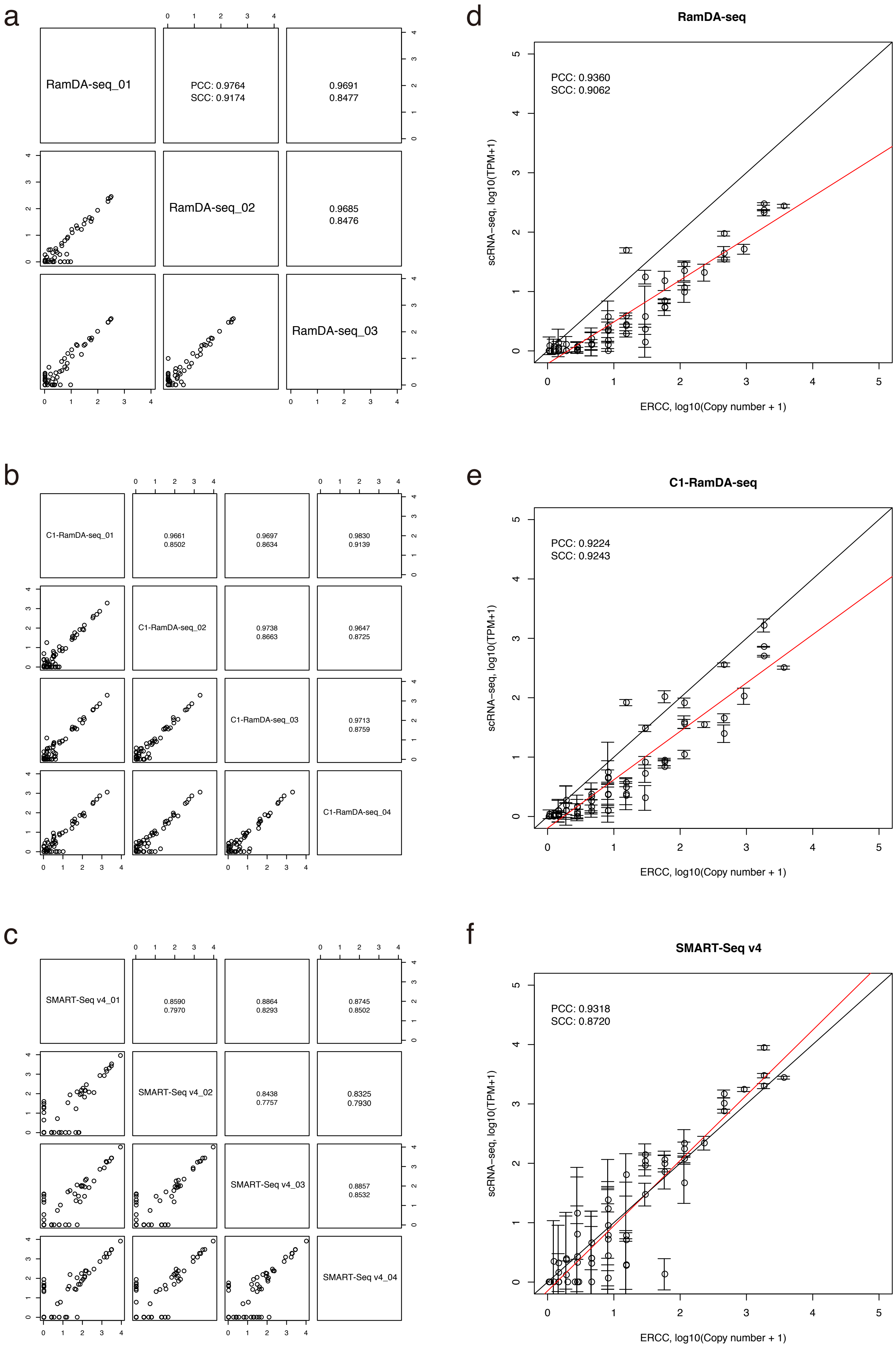
a
RamDA-seq_01
PCC: 0.9764
SCC: 0.9174
0.9691
0.8477
RamDA-seq_02
0.9685
0.8476
RamDA-seq_03
b
C1-RamDA-seq_01
0.9661
0.8502
0.9697
0.8634
0.9830
0.9139
C1-RamDA-seq_02
0.9738
0.8663
0.9647
0.8725
C1-RamDA-seq_03
0.9713
0.8759
C1-RamDA-seq_04
c
SMART-Seq v4_01
0.8590
0.7970
0.8864
0.8293
0.8745
0.8502
SMART-Seq v4_02
0.8438
0.7757
0.8325
0.7930
SMART-Seq v4_03
0.8857
0.8532
SMART-Seq v4_04
d
RamDA-seq
PCC: 0.9360
SCC: 0.9062
scRNA-seq, log10(TPM+1)
ERCC, log10(Copy number + 1)
e
C1-RamDA-seq
PCC: 0.9224
SCC: 0.9243
scRNA-seq, log10(TPM+1)
ERCC, log10(Copy number + 1)
f
SMART-Seq v4
PCC: 0.9318
SCC: 0.8720
scRNA-seq, log10(TPM+1)
ERCC, log10(Copy number + 1)

Supplementary Figure 6. Scatter plots of ERCC RNA spikes

(**a**-**c**) Scatter plot of the TPM of ERCC RNA spikes between replicate single-cell RNA-seq data using 10 pg of diluted mESC total RNA for (**a**) RamDA-seq, (**b**) C1-RamDA-seq, and (**c**) SMART-Seq v4. (**d**-**f**) Scatter plot of the TPM of ERCC RNA spikes against the numbers of input molecules (log10 (copy number + 1)) for (**d**) RamDA-seq, (**e**) C1-RamDA-seq, and (**f**) SMART-Seq v4 using 10 pg of diluted mESC total RNA. The maximum number of input molecules of an ERCC RNA spike was adjusted for 3,613 copies (ERCC-00130). The points and error bars represent means and standard deviations across replicate single-cell RNA-seq data, respectively. The black lines indicate y=x. The red lines indicate the fitted lines obtained by linear regression. The upper and lower numbers on each panel indicate the Pearson correlation coefficient (PCC) and Spearman correlation coefficient (SCC), respectively.

Percentage coverage with the absolute length of transcripts

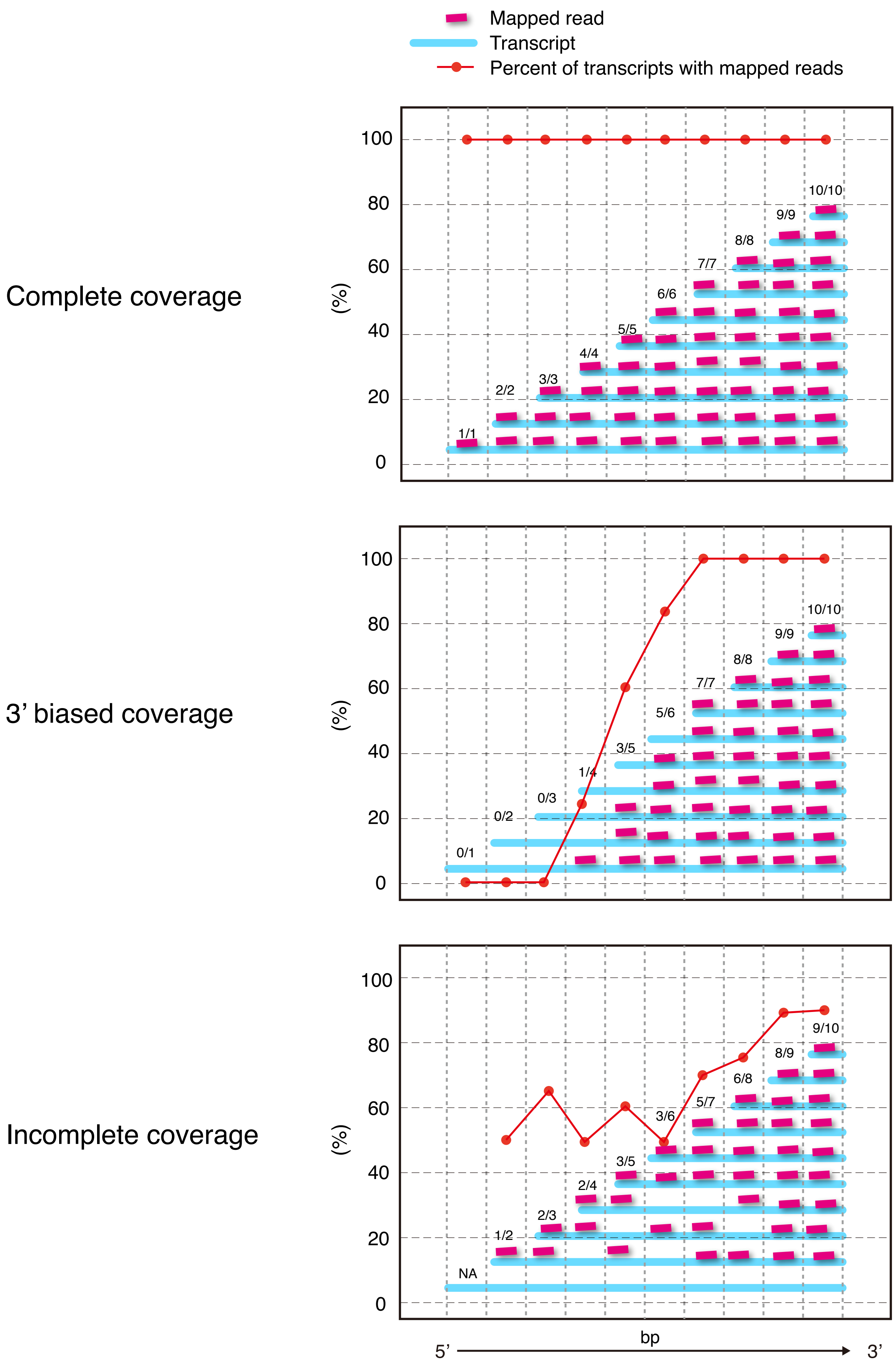

Supplementary Figure 7. Schematic diagram of the percentage coverage with the absolute length of transcripts in Figure 2a

The plots indicate three types of models for the transcript coverage, complete coverage, 3' biased coverage and incomplete coverage. The *x*-axis shows the absolute distance (bp) from the 3' end of the transcripts (x_i). The y-axis shows the fraction of transcripts with read coverage (n_i/N_i). n_i: the number of transcripts of which at least one read was mapped within the range of $[x_i, x_{i+1}]$. N_i: the number of transcripts with ≥ x_i transcript length.

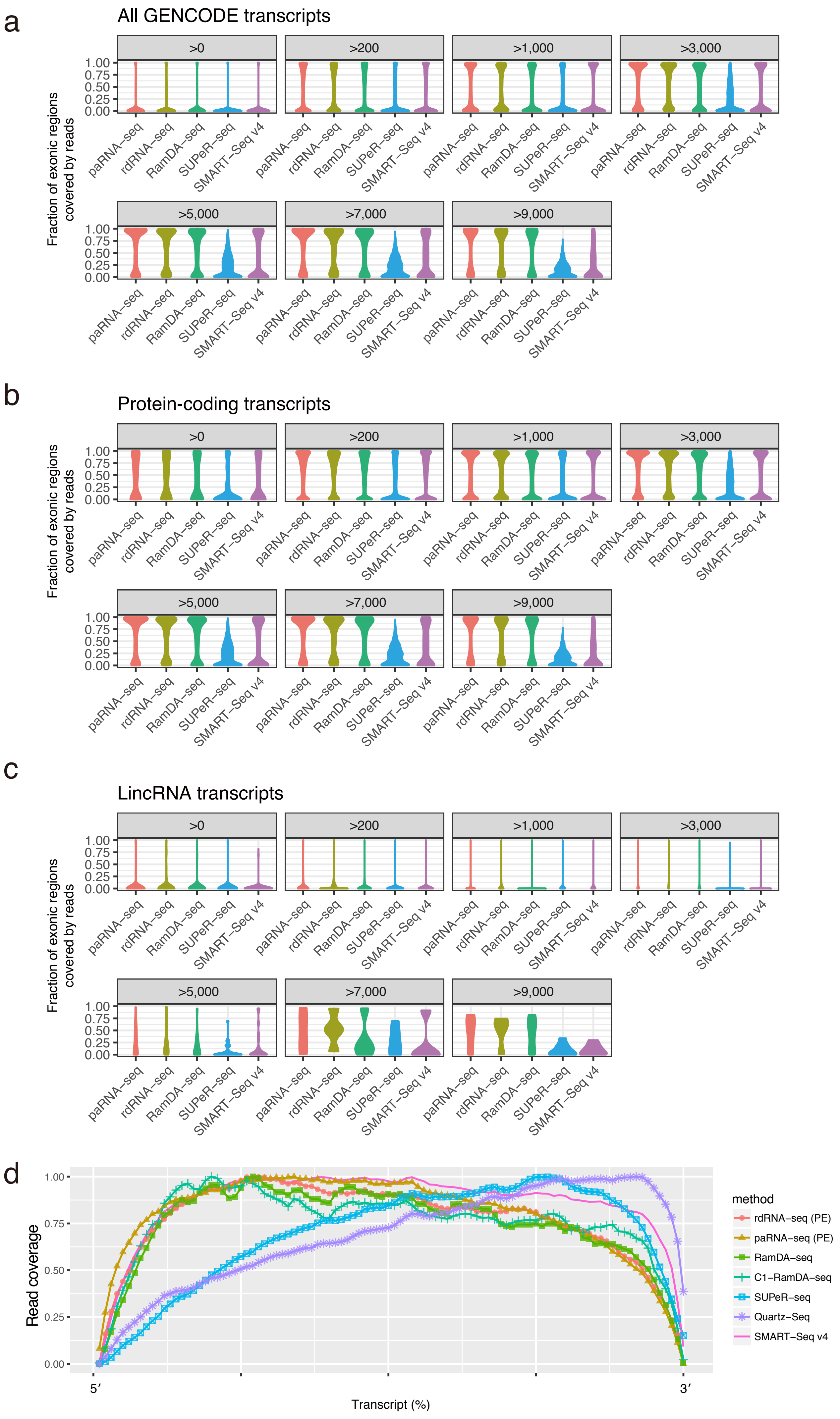

a
All GENCODE transcripts
>0
>200
>1,000
>3,000
>5,000
>7,000
>9,000
Fraction of exonic regions covered by reads
1.00
0.75
0.50
0.25
0.00
paRNA−seq
rdRNA−seq
RamDA−seq
SUPeR−seq
SMART−Seq v4
b
Protein-coding transcripts
c
LincRNA transcripts
d
Read coverage
1.00
0.75
0.50
0.25
0.00
5′
3′
Transcript (%)
method
rdRNA−seq (PE)
paRNA−seq (PE)
RamDA−seq
C1−RamDA−seq
SUPeR−seq
Quartz−Seq
SMART−Seq v4

Supplementary Figure 8. Read coverage in exonic regions

(**a**-**c**) The fraction of exonic regions covered by sequenced reads with 10 pg of RNA data for all transcripts (**a**), protein-coding transcripts (**b**), and lincRNA transcripts (**c**) in the GENCODE (vM9) annotations. The transcripts were sorted into bins (represented by the number at the top of each panel) according to transcript length. (**d**) Mean read coverage over transcripts with 10 pg of RNA data. PE: Data from paired-end reads.

ENSMUST00000097897, *Macf1* (23,495 bp)

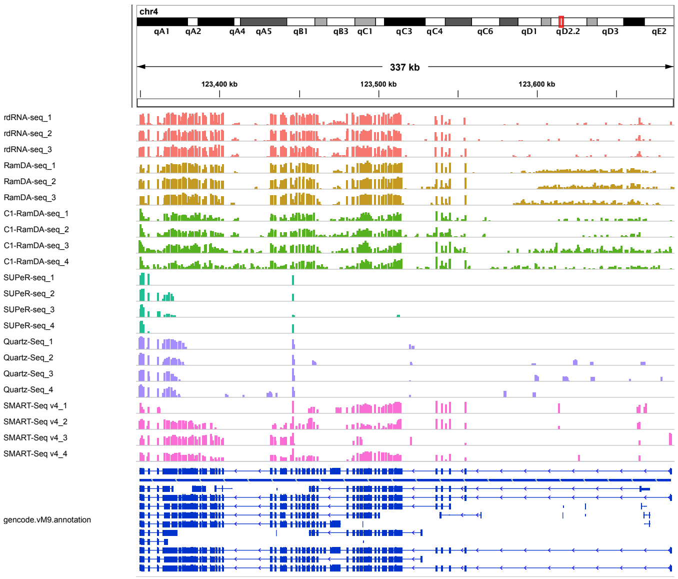

ENSMUST00000092956, *Ahnak* (18,099 bp)

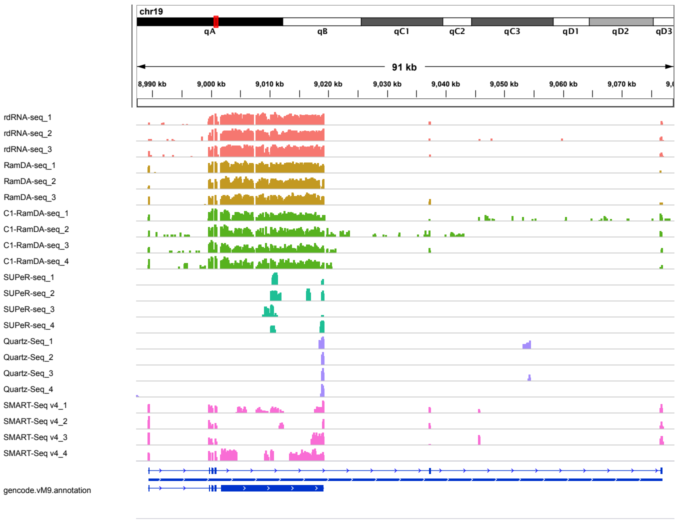

ENSMUST00000071642, *Mdn1* (17,970 bp)

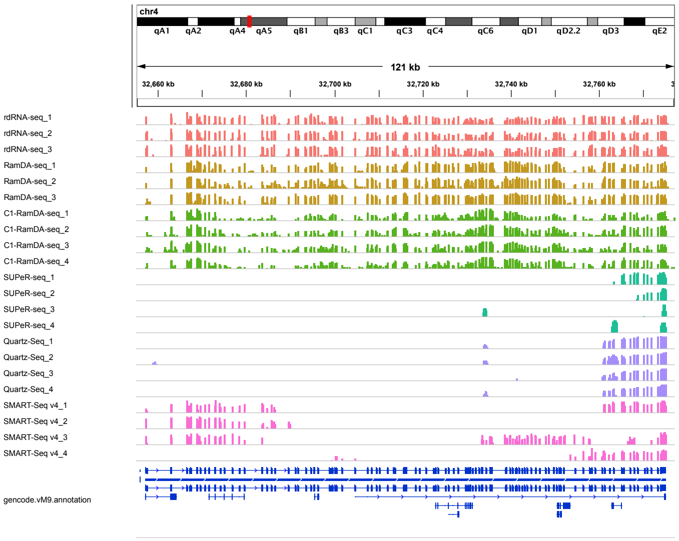

ENSMUST00000032891, *Smg1* (15,557 bp)

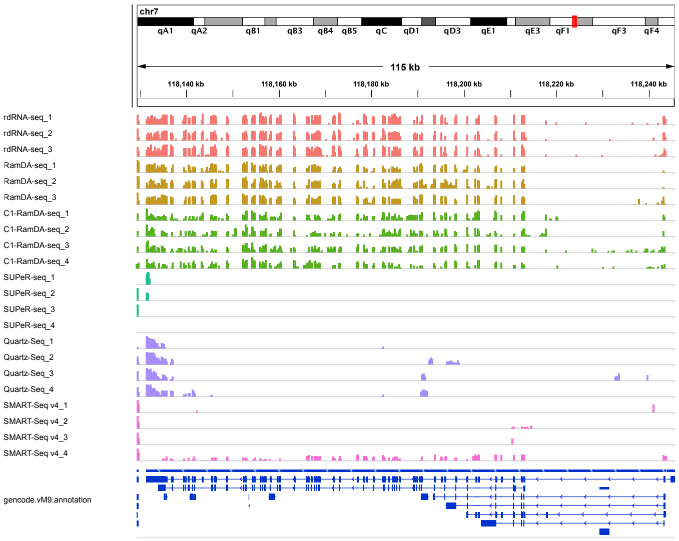

ENSMUST00000191428, *Fat1* (14,814 bp)

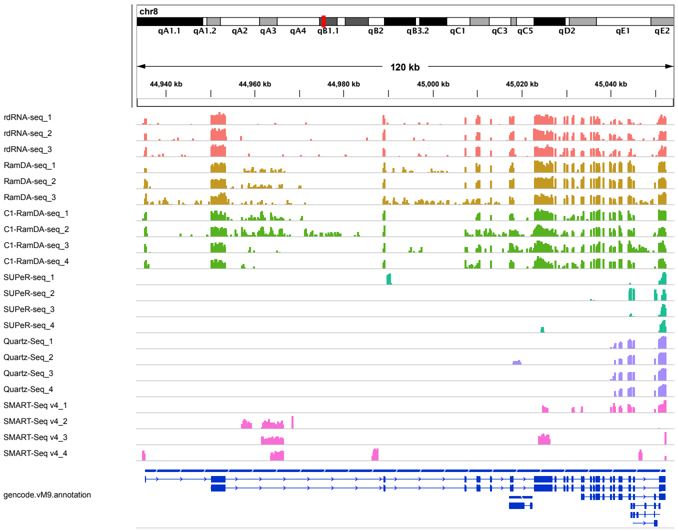

ENSMUST00000180037, *Birc6* (15,768 bp)

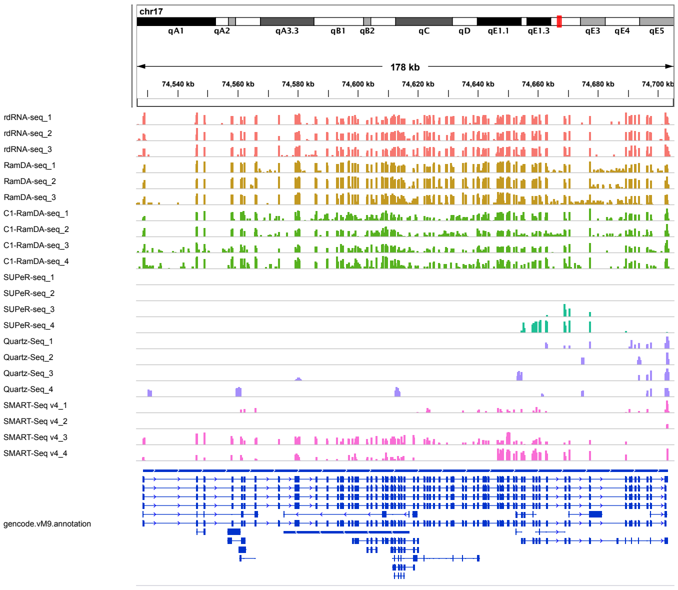

ENSMUST00000174189, *Tet1* (14,369 bp)

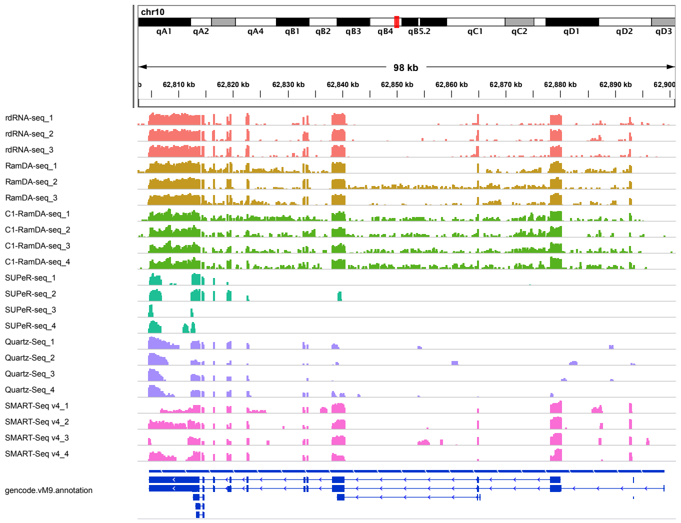

ENSMUST00000018851, *Dync1h1* (14,342 bp)

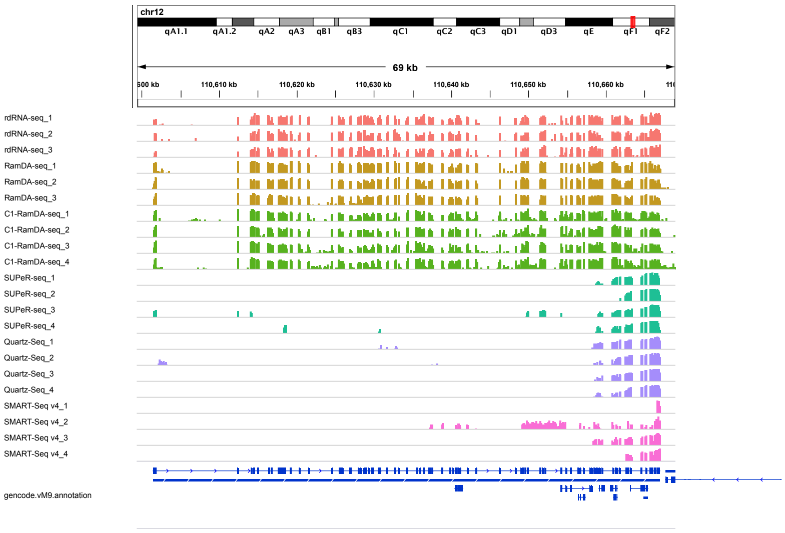

ENSMUST00000041124, *Zfp704* (13,834 bp)

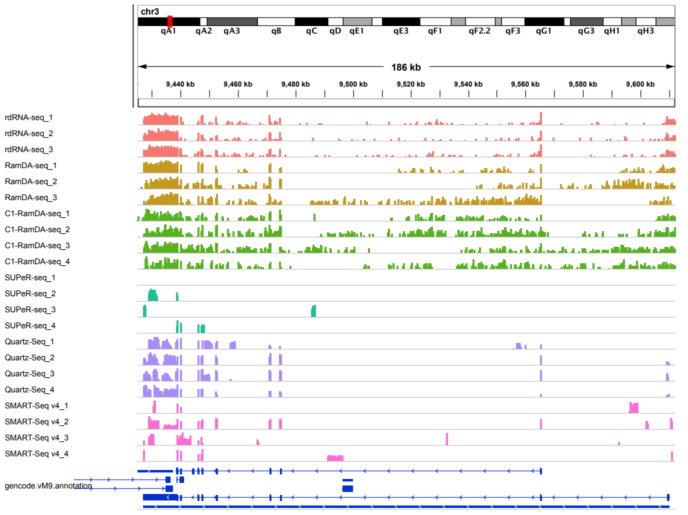

ENSMUST00000044113, *Ago2* (13,705 bp)

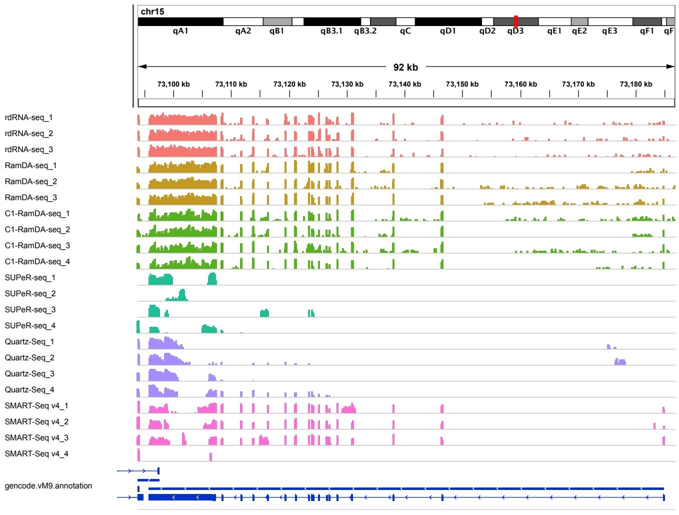

ENSMUST00000099490, *Nsd1* (12,784 bp)

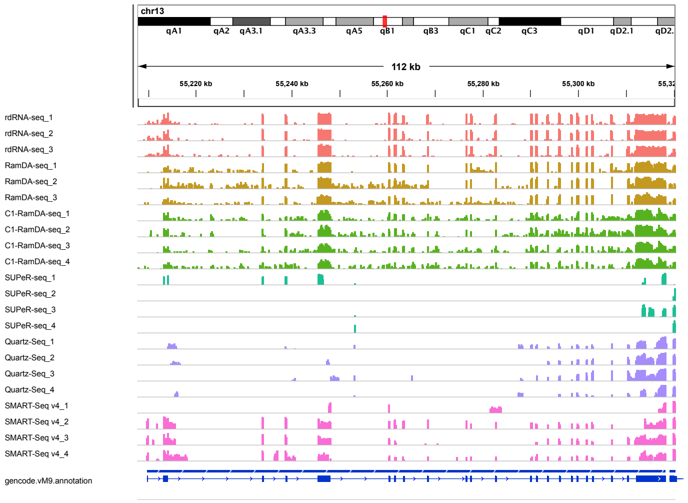

ENSMUST00000046717, *Mga* (11,982 bp)

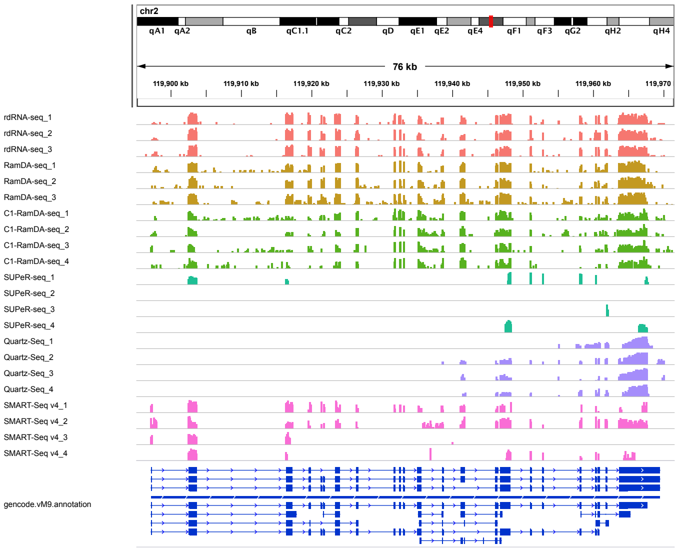

ENSMUST00000089302, *Usp9x* (11,887 bp)

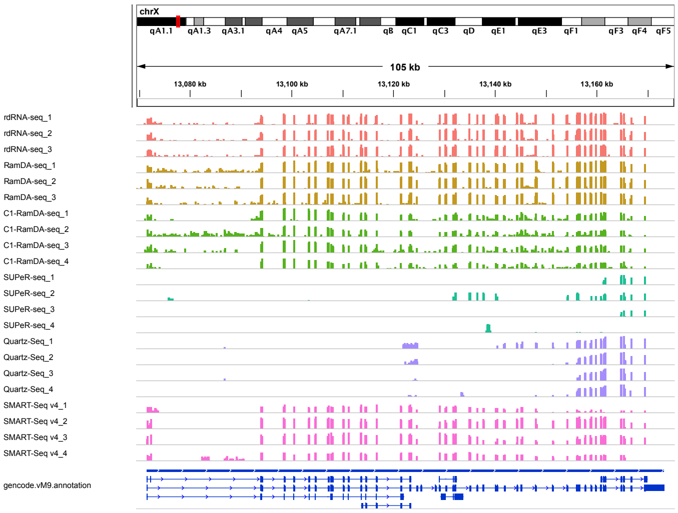

ENSMUST00000137823, *Usp34* (11,363 bp)

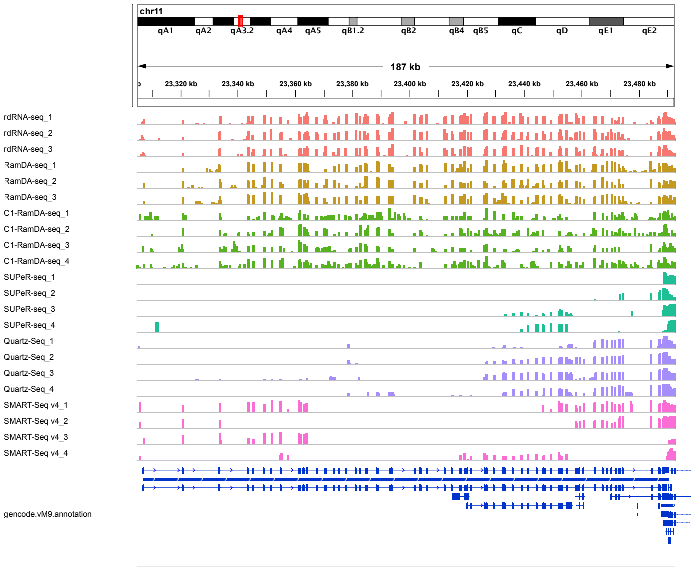

ENSMUST00000180611, *Dmxl1* (11,319 bp)

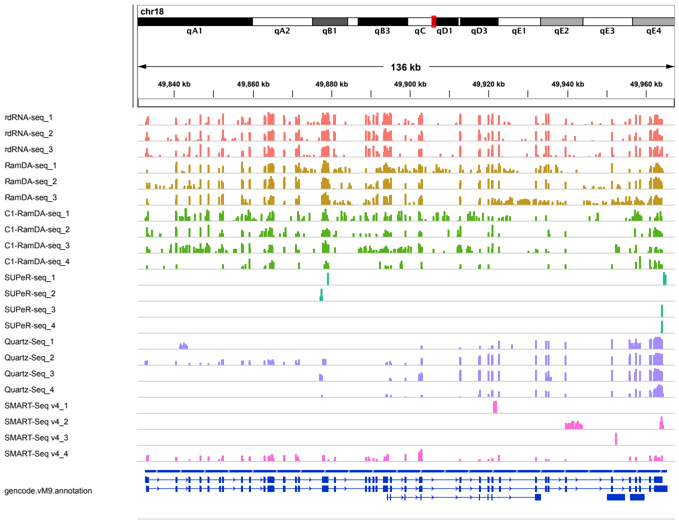

ENSMUST00000193703, *Enah* (11,229 bp)

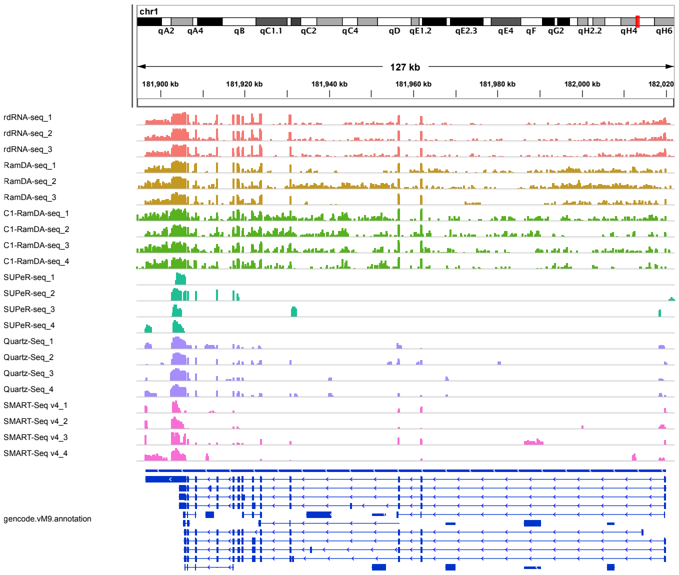

ENSMUST00000171929, *Cenpf* (11,130 bp)

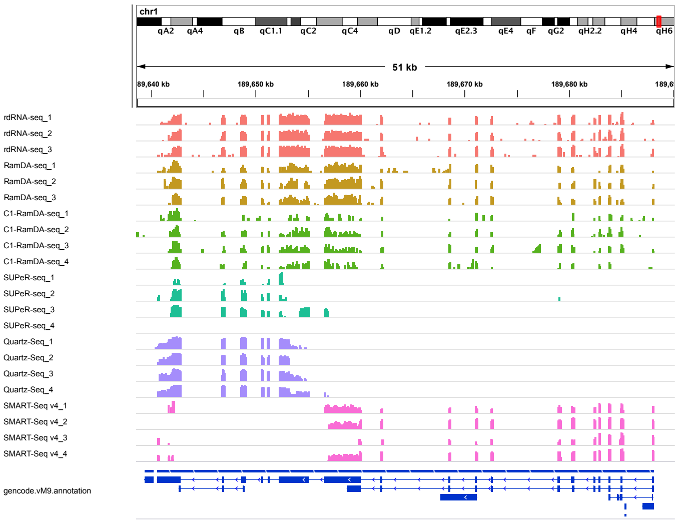

ENSMUST00000061578, *Setx* (10,970 bp)

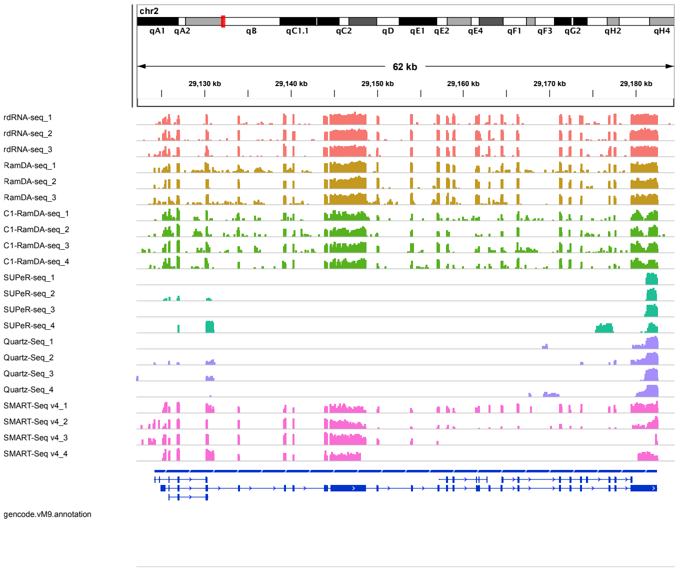

ENSMUST00000202313, *Brca2* (10,517 bp)

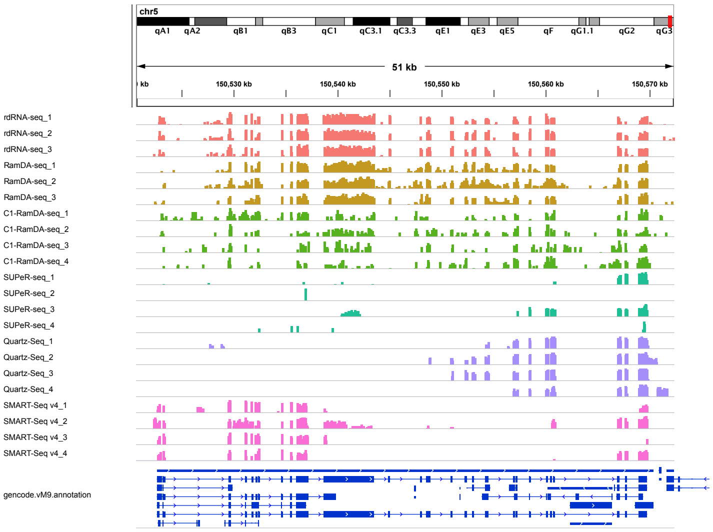

ENSMUST00000115151, *Ubxn7* (10,318 bp)

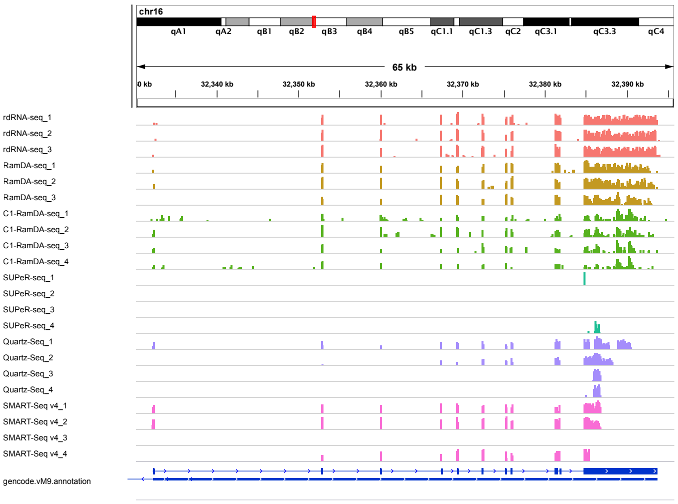

ENSMUST00000113573, *Atrx* (10,275 bp)

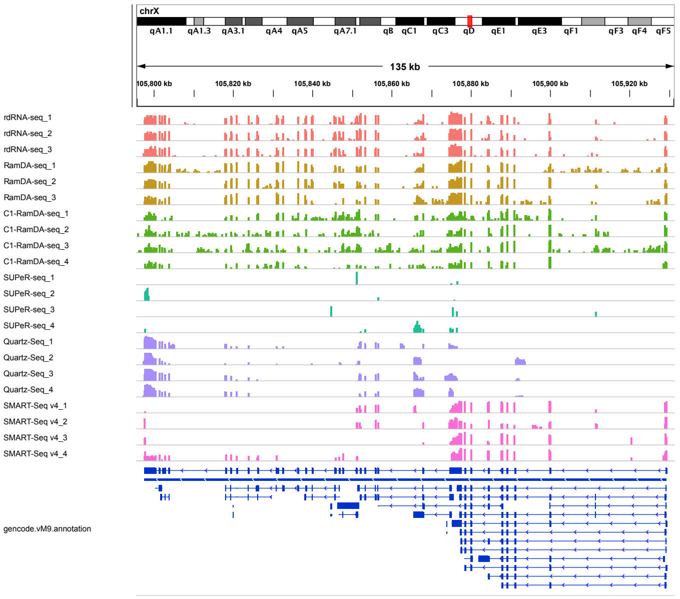

ENSMUST00000199808, *Sbno1* (10,161 bp)

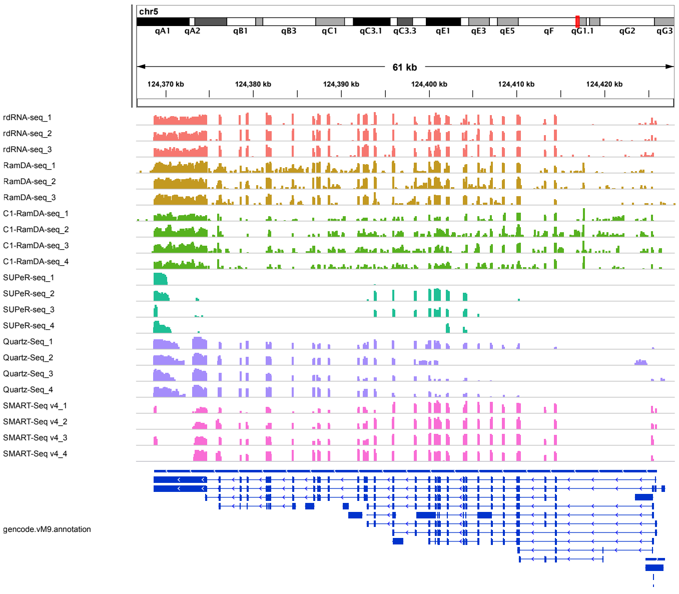

ENSMUST00000060945, *Aff4* (10,099 bp)

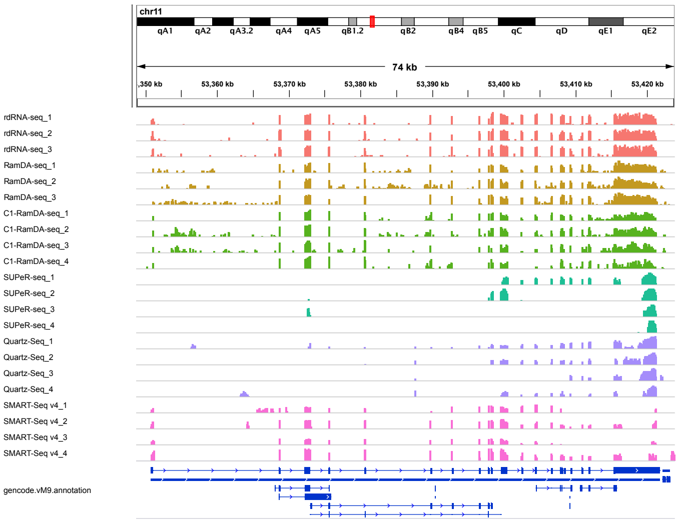

ENSMUST00000033310, *Mki67* (10,061 bp)

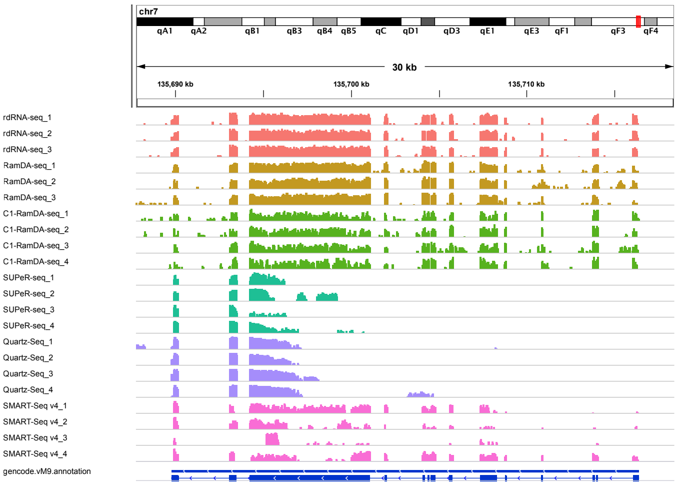

ENSMUST00000118282, *Atm* (10,006 bp)

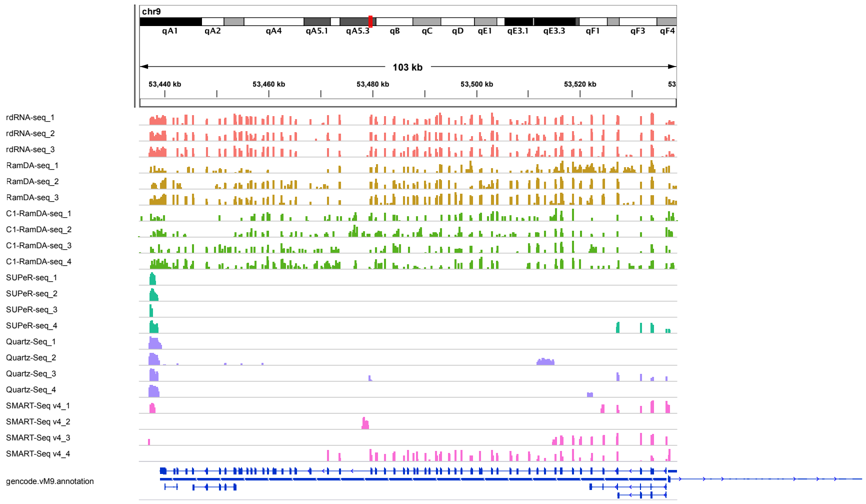

Supplementary Figure 9. Visualization and comparison of mapped reads of over 10-kb transcripts

Visualization and comparison of mapped reads of over 10-kb transcripts. The criterion for the selected 25 genes is over 10 kb in length, and the expression level is TPM > 5 in bulk rdRNA-seq.

RamDA mESC 00h, ENSMUSG00000028649.17, Macf1

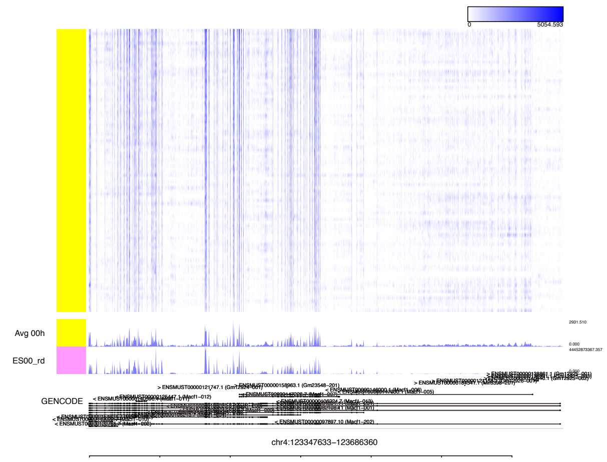

RamDA mESC 00h, ENSMUSG00000069833.12, Ahnak

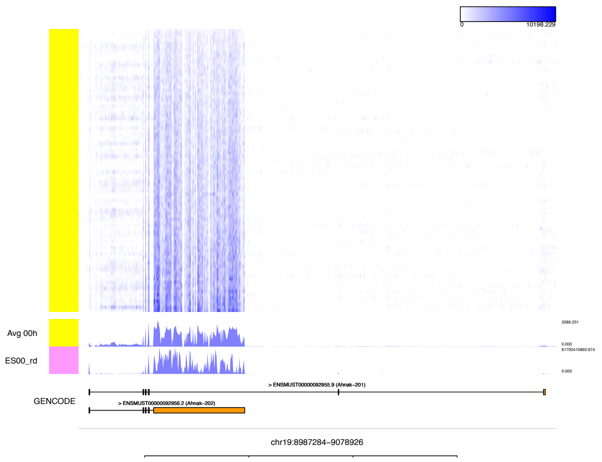

RamDA mESC 00h, ENSMUSG00000058006.12, Mdn1

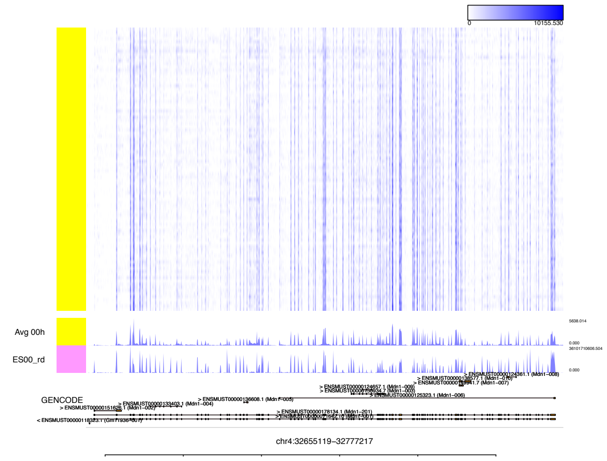

RamDA mESC 00h, ENSMUSG00000030655.15, Smg1

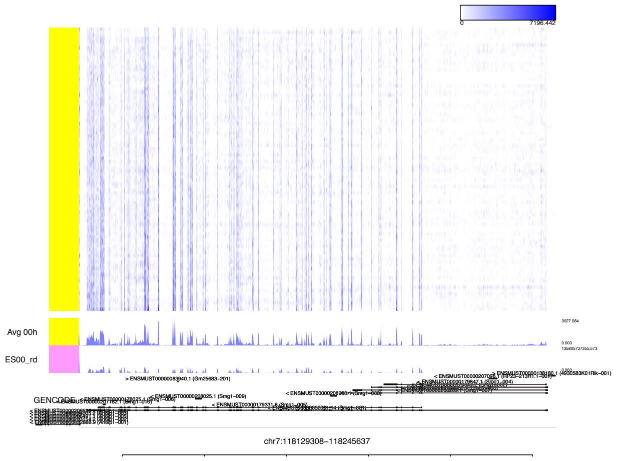

RamDA mESC 00h, ENSMUSG00000070047.12, Fat1

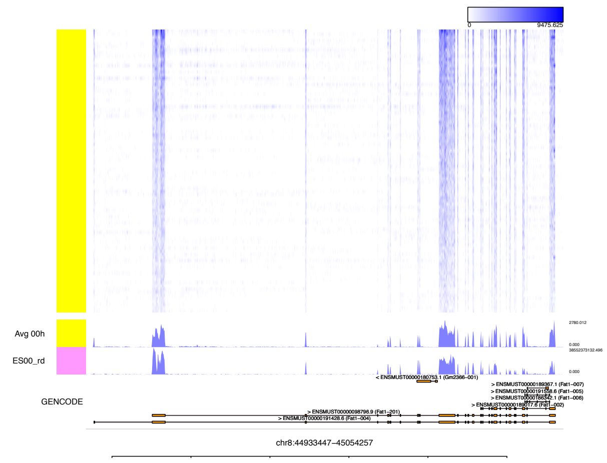

RamDA mESC 00h, ENSMUSG00000024073.14, Birc6

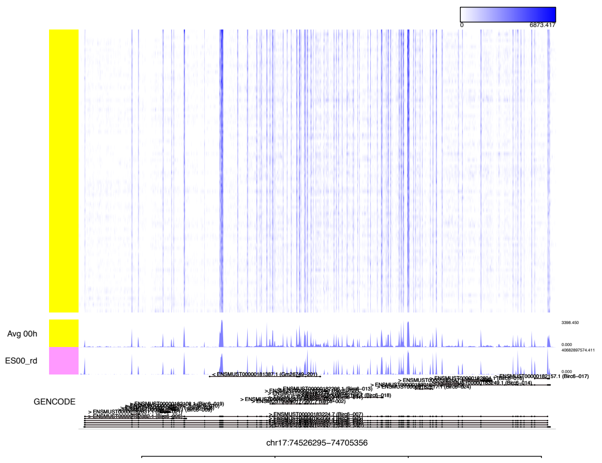

RamDA mESC 00h, ENSMUSG00000047146.15, Tet1

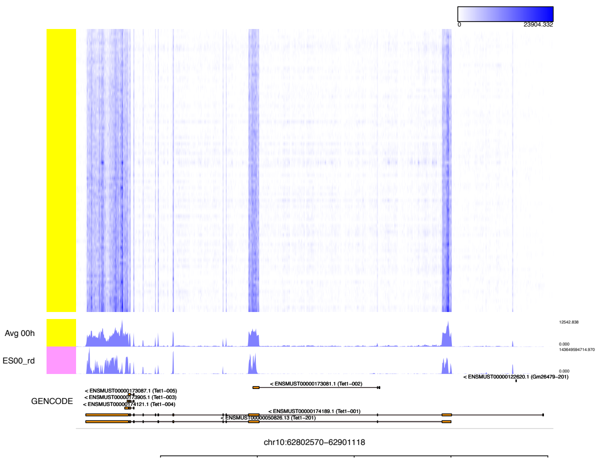

RamDA mESC 00h, ENSMUSG00000018707.13, Dync1h1

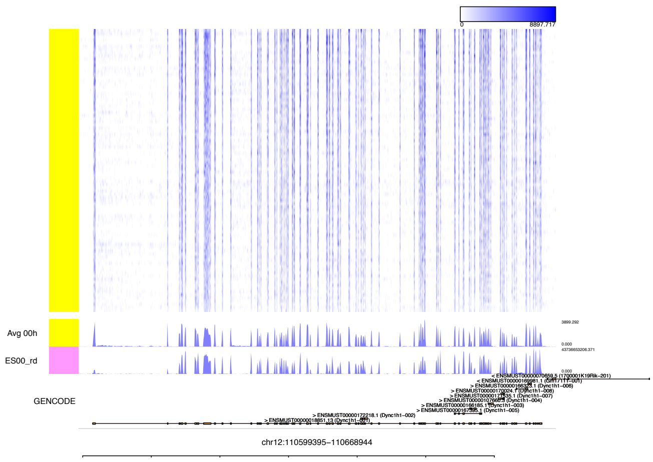

RamDA mESC 00h, ENSMUSG00000040209.12, Zfp704

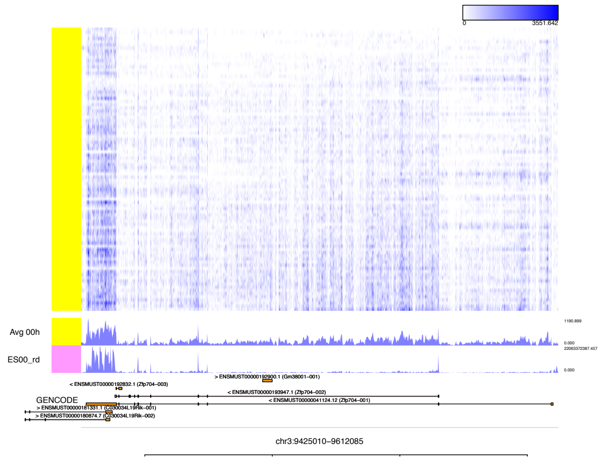

RamDA mESC 00h, ENSMUSG00000036698.10, Ago2

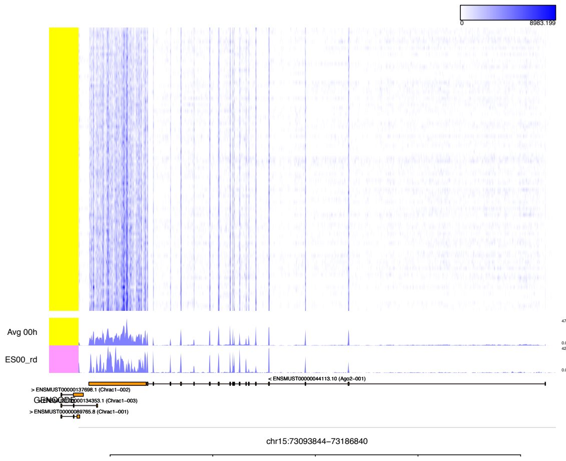

RamDA mESC 00h, ENSMUSG00000021488.7, Nsd1

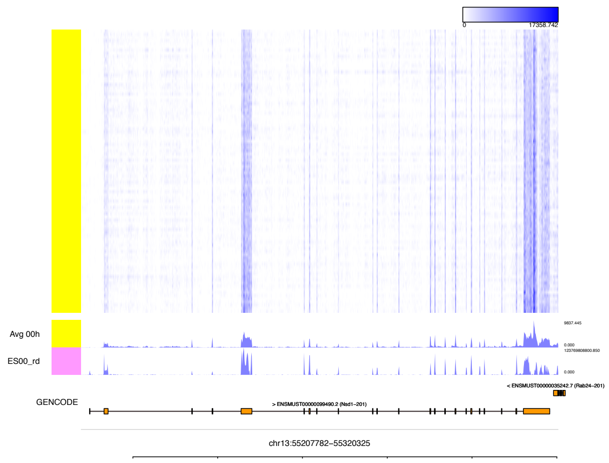

RamDA mESC 00h, ENSMUSG00000033943.15, Mga

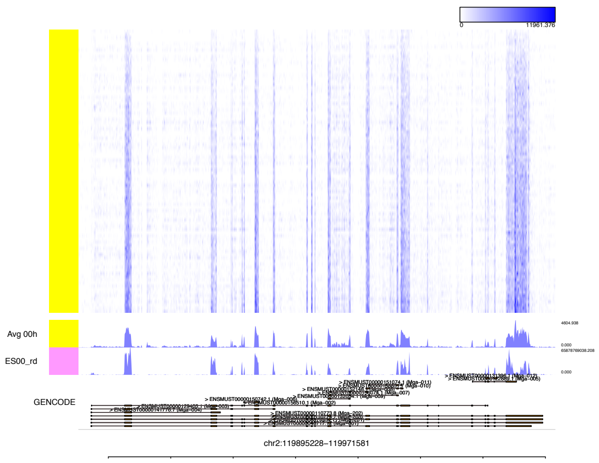

RamDA mESC 00h, ENSMUSG00000031010.17, Usp9x

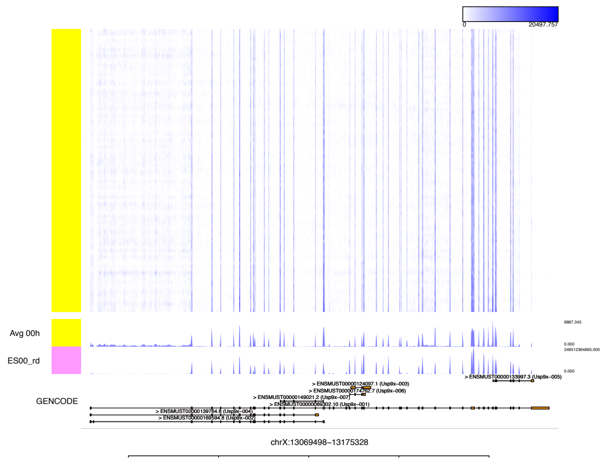

RamDA mESC 00h, ENSMUSG00000056342.16, Usp34

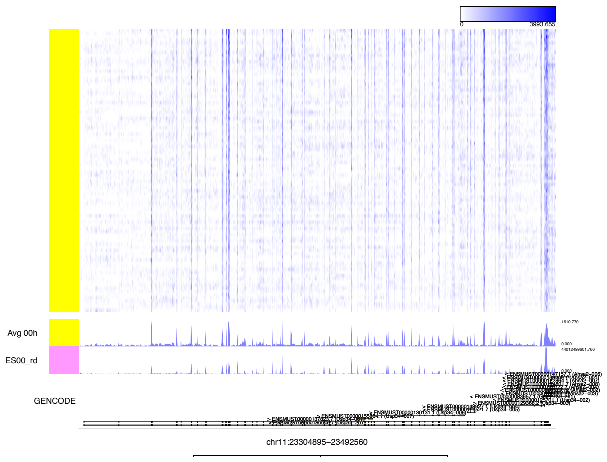

RamDA mESC 00h, ENSMUSG00000037416.12, Dmxl1

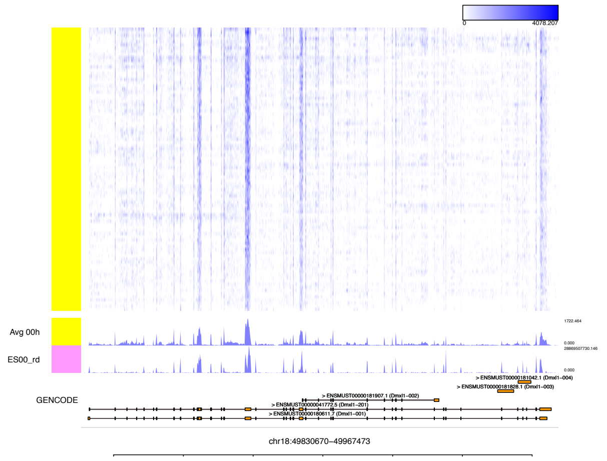

RamDA mESC 00h, ENSMUSG00000022995.16, Enah

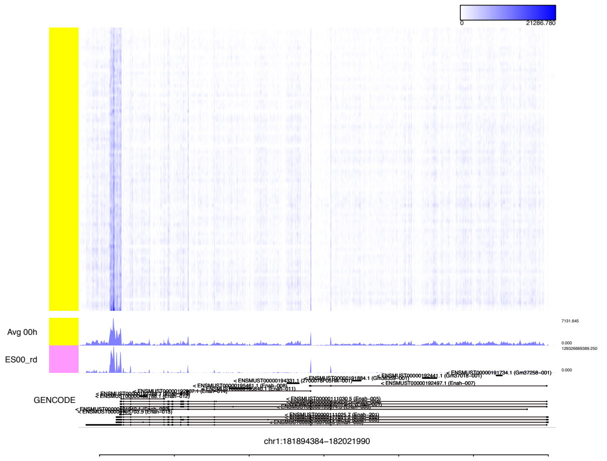

RamDA mESC 00h, ENSMUSG00000026605.14, Cenpf

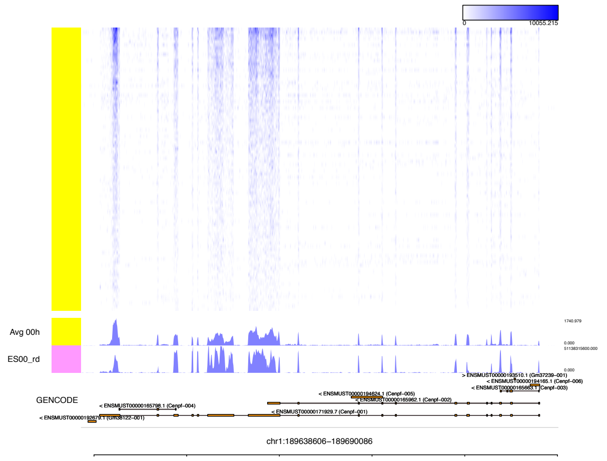

RamDA mESC 00h, ENSMUSG00000043535.13, Setx

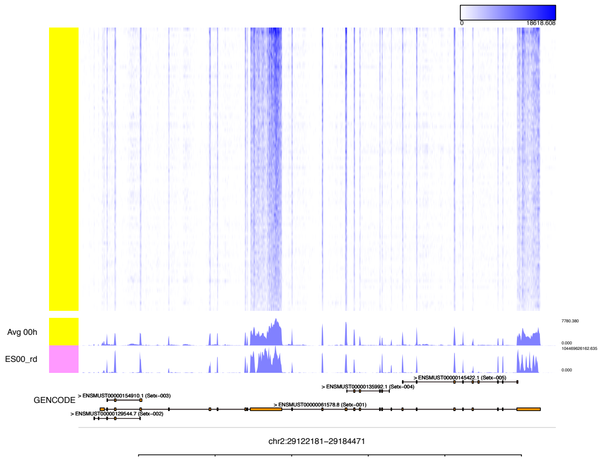

RamDA mESC 00h, ENSMUSG00000041147.10, Brca2

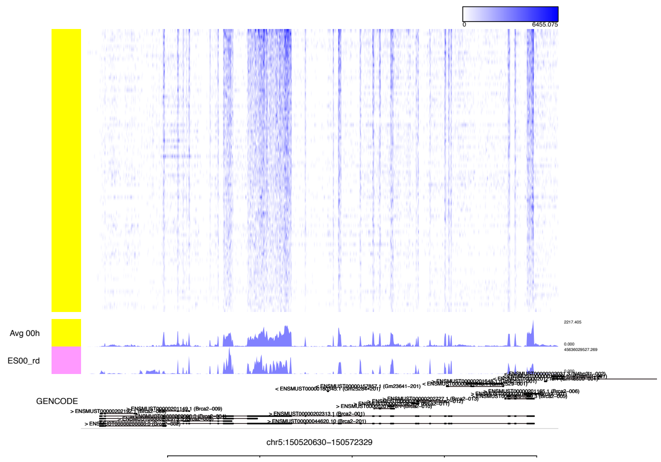

RamDA mESC 00h, ENSMUSG00000053774.8, Ubxn7

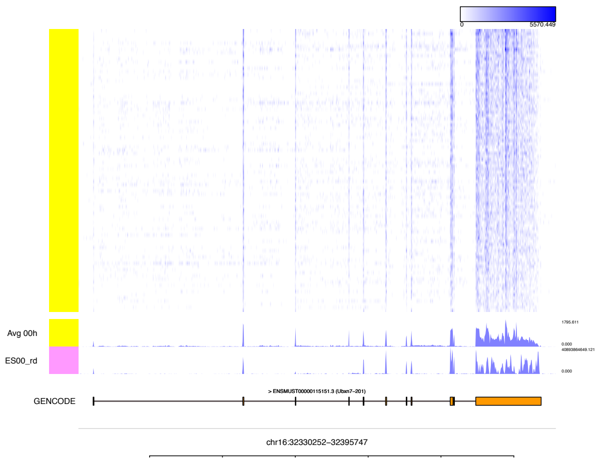

RamDA mESC 00h, ENSMUSG00000031229.16, Atrx

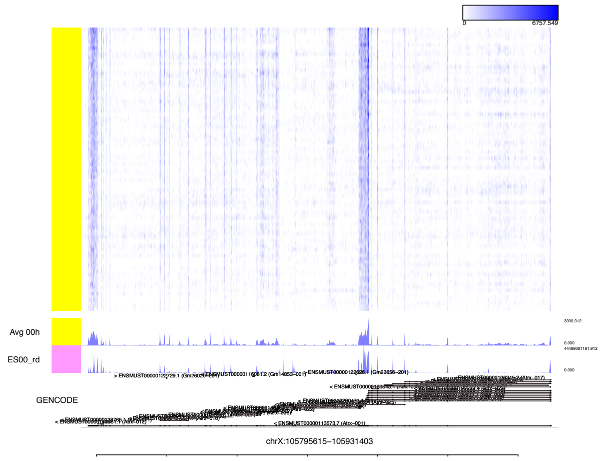

RamDA mESC 00h, ENSMUSG00000038095.15, Sbno1

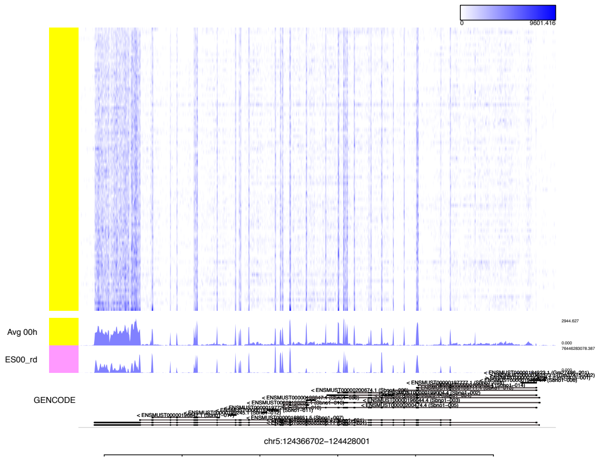

RamDA mESC 00h, ENSMUSG00000049470.13, Aff4

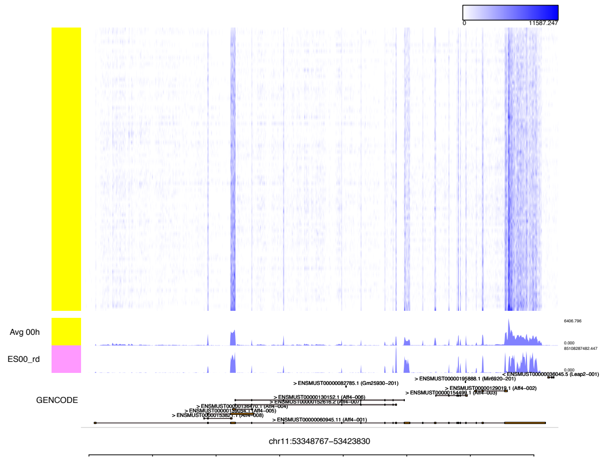

RamDA mESC 00h, ENSMUSG00000031004.7, Mki67

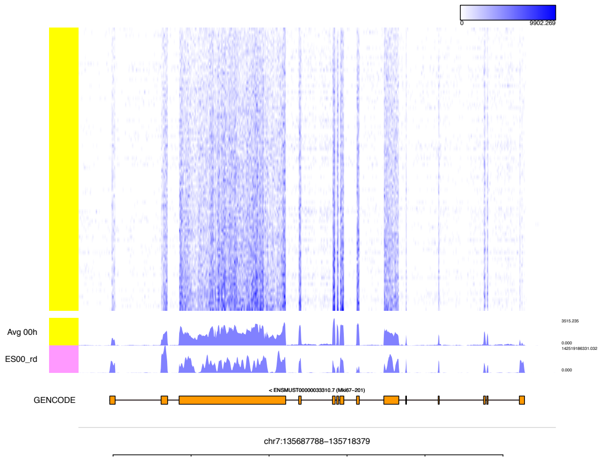

RamDA mESC 00h, ENSMUSG00000034218.15, Atm

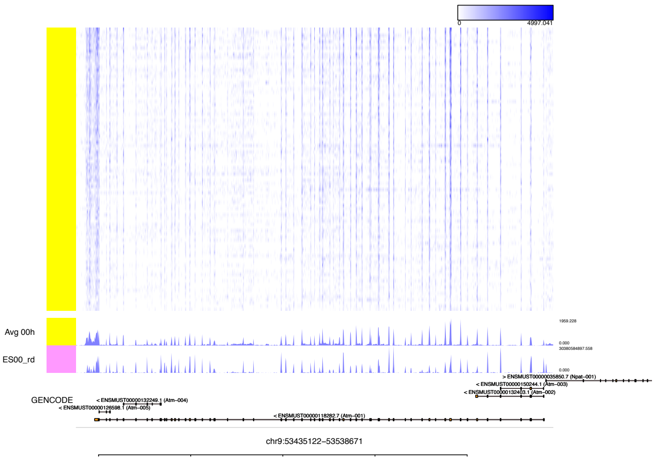

Supplementary Figure 10. Read coverages of RamDA-seq data for long transcripts in single cells

Read coverages of RamDA-seq data for long transcripts in single cells. The criterion for the selected 25 genes is over 10 kb in length, and the expression level is TPM > 5 in bulk rdRNA-seq. The upper heat map represents the read coverage of each of 90 ES cells. The middle plots represent the averaged read coverage and read coverage of rdRNA-seq (n=1). Gene models are shown at the bottom.

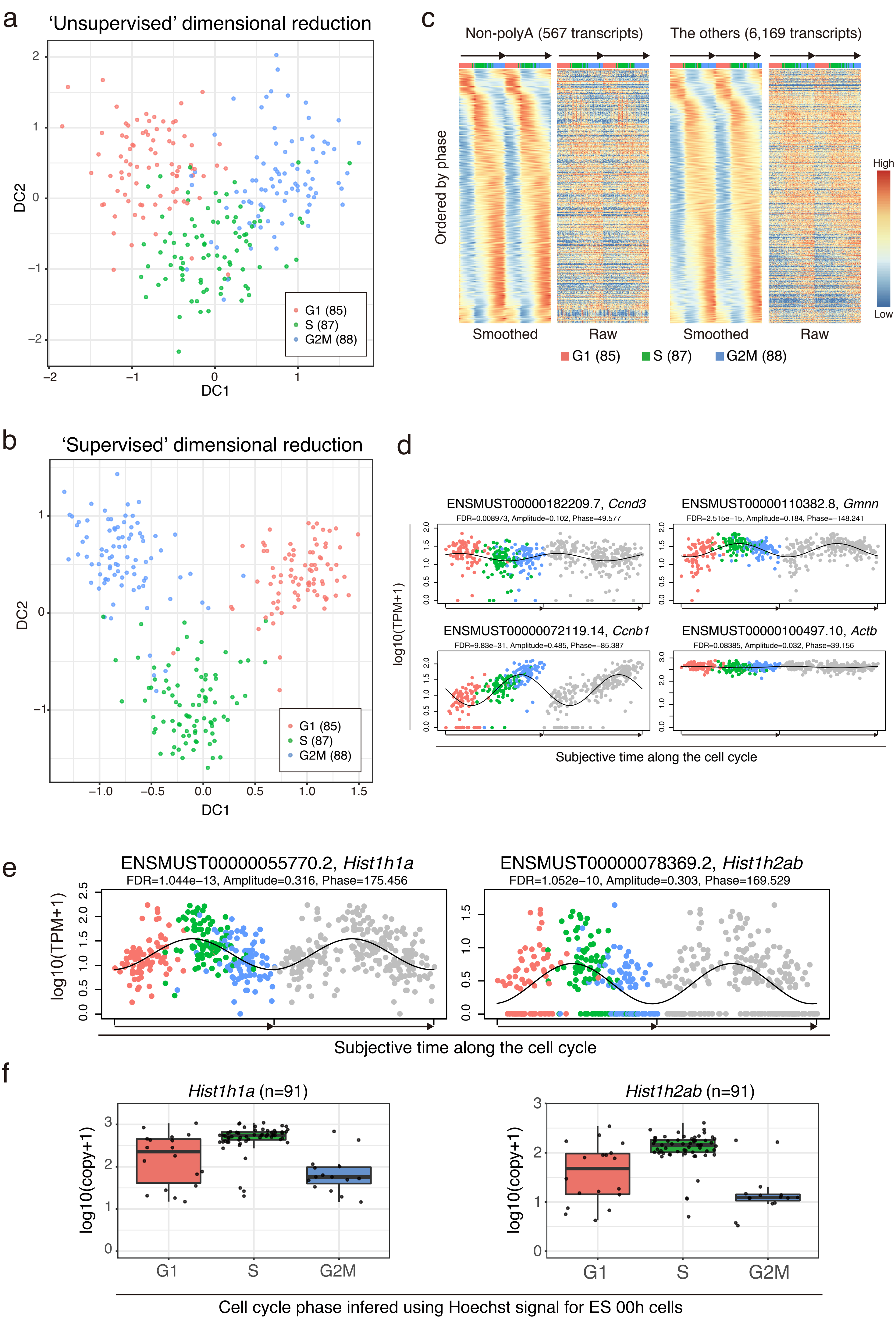
a
'Unsupervised' dimensional reduction
DC1
DC2
G1 (85)
S (87)
G2M (88)
b
'Supervised' dimensional reduction
DC1
DC2
G1 (85)
S (87)
G2M (88)
c
Non-polyA (567 transcripts)
The others (6,169 transcripts)
Ordered by phase
Smoothed
Raw
Smoothed
Raw
High
Low
G1 (85)
S (87)
G2M (88)
d
ENSMUST00000182209.7, Ccnd3
FDR=0.008973, Amplitude=0.102, Phase=49.577
ENSMUST00000110382.8, Gmnn
FDR=2.515e−15, Amplitude=0.184, Phase=−148.241
ENSMUST00000072119.14, Ccnb1
FDR=9.83e−31, Amplitude=0.485, Phase=−85.387
ENSMUST00000100497.10, Actb
FDR=0.08385, Amplitude=0.032, Phase=39.156
log10(TPM+1)
Subjective time along the cell cycle
e
ENSMUST00000055770.2, Hist1h1a
FDR=1.044e−13, Amplitude=0.316, Phase=175.456
ENSMUST00000078369.2, Hist1h2ab
FDR=1.052e−10, Amplitude=0.303, Phase=169.529
log10(TPM+1)
Subjective time along the cell cycle
f
Hist1h1a (n=91)
Hist1h2ab (n=91)
log10(copy+1)
G1
S
G2M
Cell cycle phase infered using Hoechst signal for ES 00h cells

Supplementary Figure 11. RamDA-seq analyses of the cell cycle

(**a**) An 'unsupervised' diffusion map of cell-cycle data using highly variable transcripts across all cells (see Methods). Each point represents a cell colored according to the derived cell-cycle phase. DC, diffusion component. (**b**) A 'supervised', cell-cycle phase-aware diffusion map of cell-cycle data using highly variable transcripts among cell-cycle phase (see Methods). (**c**) Heat maps of expression levels of non-poly(A) (left) and other (right) transcripts. Rows are ordered by the inferred phase. Columns are ordered by pseudotime and colored according to the derived cell-cycle phase. Smoothed values were transformed to Z-scores for each row. Raw values were scaled from 0 to 1 for each row. Columns were duplicated for visualization (double plot). (**a-c**) The numbers in parentheses represent the number of cells. (**d**) The expression profiles of cell-cycle markers (*Ccnd3*, *Gmnn*, and *Ccnb1*) and housekeeping transcripts (*Actb*) measured by RamDA-seq. The x-axis represents pseudotime. The y-axis represents expression level. Each point represents a cell colored according to the derived phase. The black curve represents the fitted sine function for each transcript. The data are shown in triplicate for clearer visualization. (**e**) Expression profiles of transcripts encoding histone proteins measured by RamDA-seq. Axes and color coding are the same as those in (d). (**f**) Expression profiles of histone-coding transcripts measured by single-cell preamplification RT-qPCR. Cells were derived from the time course sampling at 0 h and assigned to the G1, S, and G2M phases based on the Hoechst intensity determined by a cell sorter. The center line, lower and upper bounds of each box represent the median, first and third quartiles, respectively. The lower (upper) whisker extends to smallest (largest) values no further than 1.5 * IQR from the first (third) quartile.

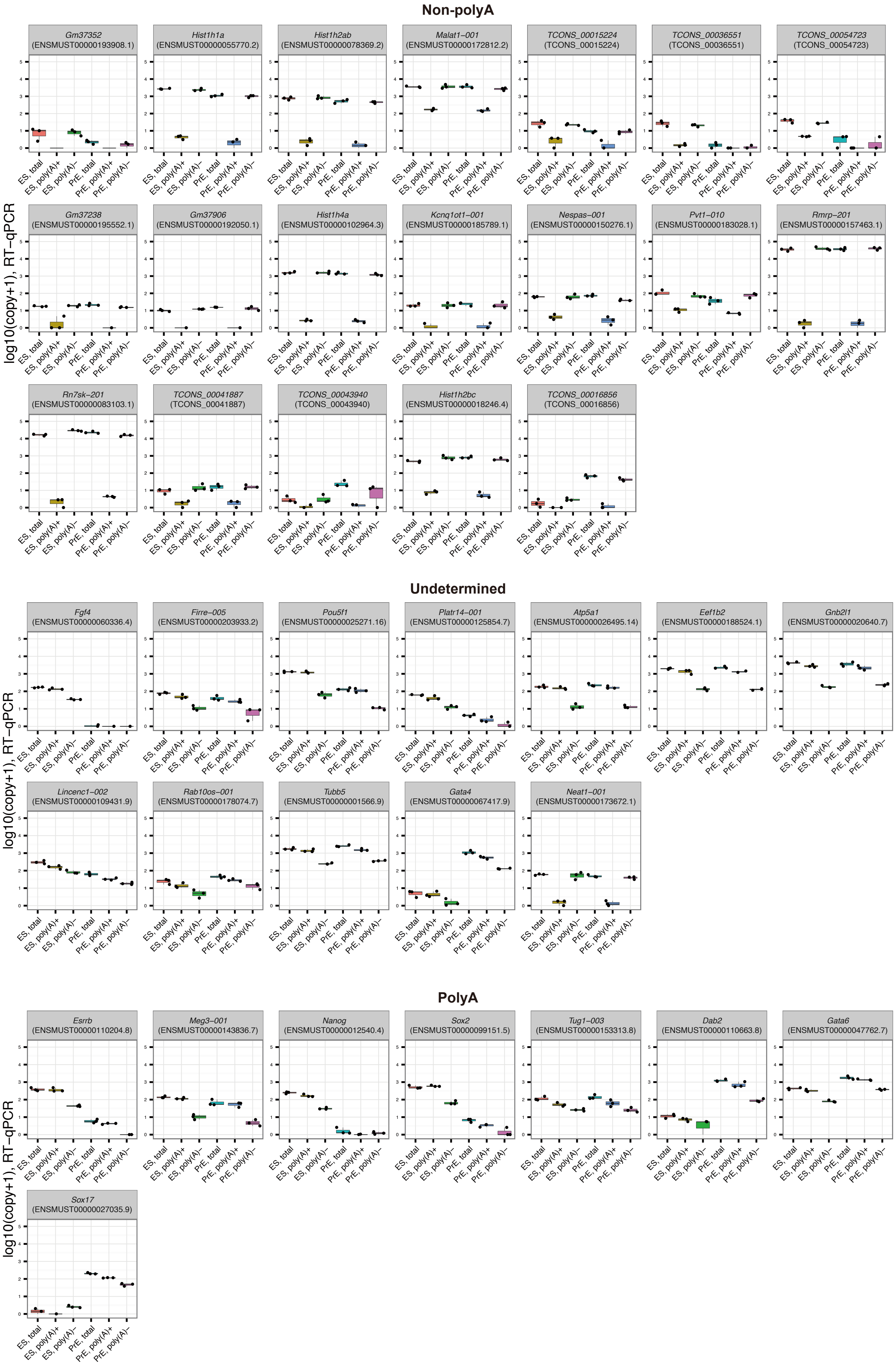
Non-polyA
Gm37352 (ENSMUST00000193908.1)
Hist1h1a (ENSMUST00000055770.2)
Hist1h2ab (ENSMUST00000078369.2)
Malat1−001 (ENSMUST00000172812.2)
TCONS_00015224 (TCONS_00015224)
TCONS_00036551 (TCONS_00036551)
TCONS_00054723 (TCONS_00054723)
Gm37238 (ENSMUST00000195552.1)
Gm37906 (ENSMUST00000192050.1)
Hist1h4a (ENSMUST00000102964.3)
Kcnq1ot1−001 (ENSMUST00000185789.1)
Nespas−001 (ENSMUST00000150276.1)
Pvt1−010 (ENSMUST00000183028.1)
Rmrp−201 (ENSMUST00000157463.1)
Rn7sk−201 (ENSMUST00000083103.1)
TCONS_00041887 (TCONS_00041887)
TCONS_00043940 (TCONS_00043940)
Hist1h2bc (ENSMUST00000018246.4)
TCONS_00016856 (TCONS_00016856)
Undetermined
Fgf4 (ENSMUST00000060336.4)
Firre−005 (ENSMUST00000203933.2)
Pou5f1 (ENSMUST00000025271.16)
Platr14−001 (ENSMUST00000125854.7)
Atp5a1 (ENSMUST00000026495.14)
Eef1b2 (ENSMUST00000188524.1)
Gnb2l1 (ENSMUST00000020640.7)
Lincenc1−002 (ENSMUST00000109431.9)
Rab10os−001 (ENSMUST00000178074.7)
Tubb5 (ENSMUST00000001566.9)
Gata4 (ENSMUST00000067417.9)
Neat1−001 (ENSMUST00000173672.1)
PolyA
Esrrb (ENSMUST00000110204.8)
Meg3−001 (ENSMUST00000143836.7)
Nanog (ENSMUST00000012540.4)
Sox2 (ENSMUST00000099151.5)
Tug1−003 (ENSMUST00000153313.8)
Dab2 (ENSMUST00000110663.8)
Gata6 (ENSMUST00000047762.7)
Sox17 (ENSMUST00000027035.9)
log10(copy+1), RT−qPCR
ES, total
ES, poly(A)+
ES, poly(A)−
PrE, total
PrE, poly(A)+
PrE, poly(A)−

Supplementary Figure 12. Experimental validation of non-poly(A) RNAs using RT-qPCR

RT-qPCR analysis of non-poly(A) and poly(A) RNAs. Each point represents the log10 (copy number + 1) in the total, poly(A)(+), and poly(A)(-) fractions in ES and PrE cells. The center line, lower and upper bounds of each box represent the median, first and third quartiles, respectively. The lower (upper) whisker extends to smallest (largest) values no further than 1.5 * IQR from the first (third) quartile.

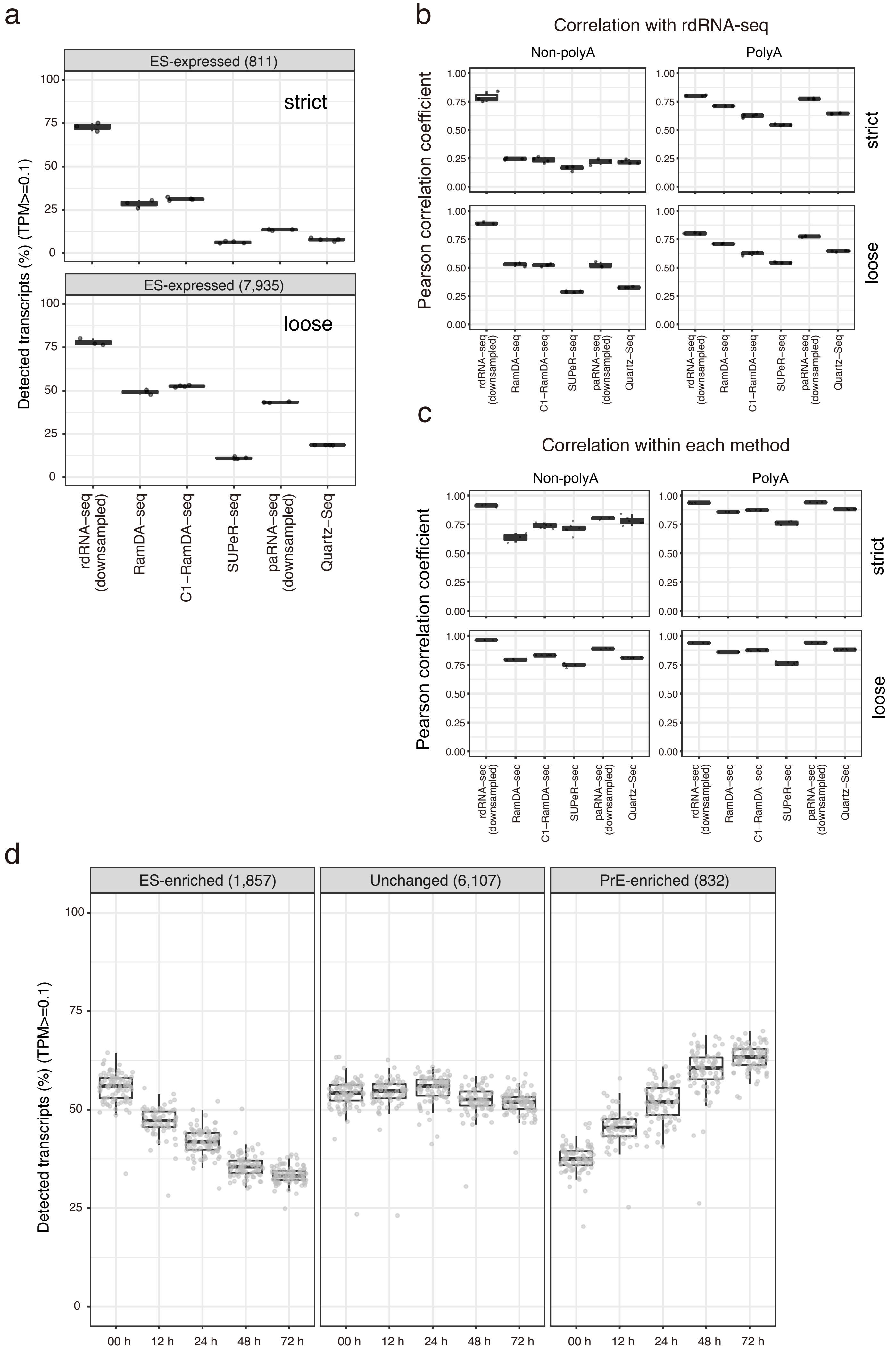
a
ES-expressed (811)
strict
ES-expressed (7,935)
loose
Detected transcripts (%) (TPM>=0.1)
100
75
50
25
0
rdRNA−seq (downsampled)
RamDA−seq
C1−RamDA−seq
SUPeR−seq
paRNA−seq (downsampled)
Quartz−Seq
b
Correlation with rdRNA-seq
Non-polyA
PolyA
strict
loose
Pearson correlation coefficient
1.00
0.75
0.50
0.25
0.00
c
Correlation within each method
Non-polyA
PolyA
strict
loose
Pearson correlation coefficient
d
ES-enriched (1,857)
Unchanged (6,107)
PrE-enriched (832)
Detected transcripts (%) (TPM>=0.1)
00 h
12 h
24 h
48 h
72 h

Supplementary Figure 13. Sensitivity to non-poly(A) transcripts for different scRNA-seq methods

(**a**) Sensitivity analysis of scRNA-seq methods for detecting non-poly(A) transcripts expressed in ESCs with strict (top) and loose (bottom) criteria. Each point represents the fraction of the detected non-poly(A) transcripts per sample. The numbers in parentheses represent the number of transcripts. (**b**) Pearson correlation coefficients of the expression levels of non-poly(A) (left; strict (top) and loose (bottom) criteria) and poly(A) (right; loose criterion) transcripts between rdRNA-seq and each scRNA-seq method. Only transcripts with ≥ 0.1 TPM in rdRNA-seq were considered. (**c**) Pearson correlation coefficients of the expression levels of non-poly(A) (left; strict (top) and loose (bottom) criteria) and poly(A) (right; loose criterion) transcripts within each scRNA-seq method. Only transcripts with ≥ 0.1 TPM in rdRNA-seq were considered. (**d**) Sensitivity analysis of the detection of non-poly(A) transcripts with loose criteria for RamDA-seq data with living cells. Each panel represents non-poly(A) transcripts enriched in ESCs (left) and PrE cells (right) or transcripts that remained unchanged between the two cell types (middle). The *y*-axis represents the fraction of detected non-poly(A) transcripts. The numbers in parentheses represent the number of transcripts. (**a-d**) The center line, lower and upper bounds of each box represent the median, first and third quartiles, respectively. The lower (upper) whisker extends to smallest (largest) values no further than 1.5 * IQR from the first (third) quartile.

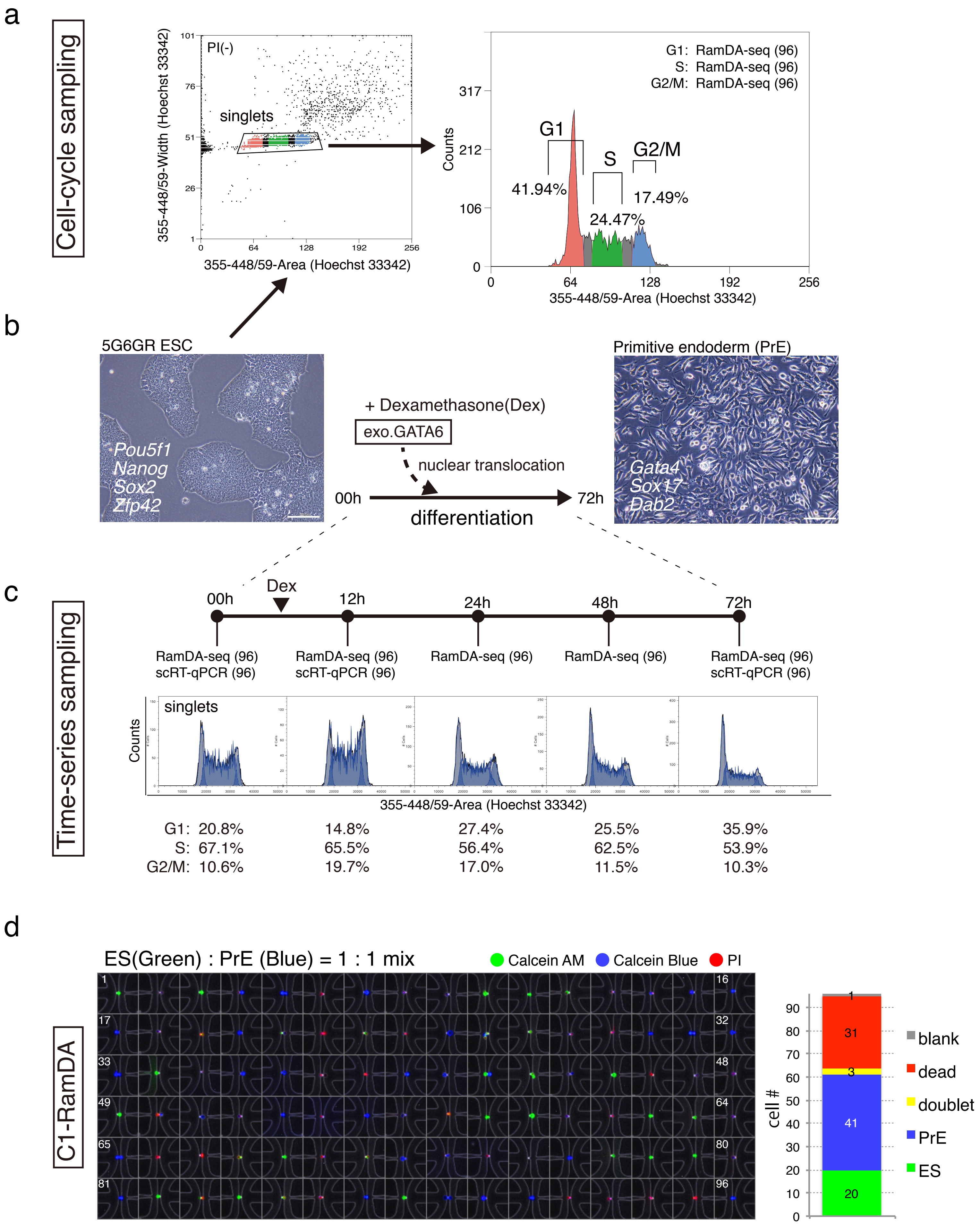
a
Cell-cycle sampling
PI(-)
singlets
355-448/59-Width (Hoechst 33342)
355-448/59-Area (Hoechst 33342)
G1: RamDA-seq (96)
S: RamDA-seq (96)
G2/M: RamDA-seq (96)
Counts
G1
S
G2/M
41.94%
24.47%
17.49%
b
5G6GR ESC
Pou5f1
Nanog
Sox2
Zfp42
+ Dexamethasone(Dex)
exo.GATA6
nuclear translocation
00h
72h
differentiation
Primitive endoderm (PrE)
Gata4
Sox17
Dab2
c
Time-series sampling
00h
Dex
12h
24h
48h
72h
RamDA-seq (96)
scRT-qPCR (96)
RamDA-seq (96)
scRT-qPCR (96)
RamDA-seq (96)
RamDA-seq (96)
RamDA-seq (96)
scRT-qPCR (96)
singlets
Counts
355-448/59-Area (Hoechst 33342)
G1: 20.8% 14.8% 27.4% 25.5% 35.9%
S: 67.1% 65.5% 56.4% 62.5% 53.9%
G2/M: 10.6% 19.7% 17.0% 11.5% 10.3%
d
C1-RamDA
ES(Green) : PrE (Blue) = 1 : 1 mix
Calcein AM
Calcein Blue
PI
cell #
blank
dead
doublet
PrE
ES
1
31
3
41
20

Supplementary Figure 14. Experimental design of the RamDA-seq and C1-RamDA-seq applications

(**a**) Cell-cycle sampling. Singlet cell-cell fractions were defined using the Hoechst 33342-Area vs Hoechst 33342-Width in PI-minus gated cells (left). After singlet cells were selected, the Hoechst intensity was used to define the fraction of cell-cycle phases (G1, S, G2/M) (right). The numbers in parentheses represent the number of collected single cells. (**b**) Differentiation process from ES to PrE cells. The 5G6GR ES cells (phase imaging, left) were added together with dexamethasone to translocate exogenous GATA6 into the nucleus, which resulted in differentiation into PrE cells (phase imaging, right). The cells were cultured for 72 h. Scale bar: 100 μm. (**c**) Time-series sampling. (Top) Cells were collected before (0 h) and 12, 24, 48, and 72 h after the addition of dexamethasone. The numbers in parentheses represent the number of collected single cells. (Bottom) Distribution of the Hoechst 33342 intensity in cells at each time point. The numbers represent the cell-cycle percentage estimated by the Watson Pragmatic algorithm. The numbers in parentheses represent the number of cells. (**d**) Quality check of cell capturing by Fluidigm C1. (Left) Image of capture sites in the integrated fluidic circuit (IFC) using a fluorescence microscope. Green (Calcein AM) and blue (Calcein Blue AM) fluorescence represent ES and PrE cells, respectively. Red (PI) fluorescence indicates dead cells. The numbers of cells indicate the cell capture site using IFC. (Right) Results of the quality assessment for cell capturing by visual inspection.

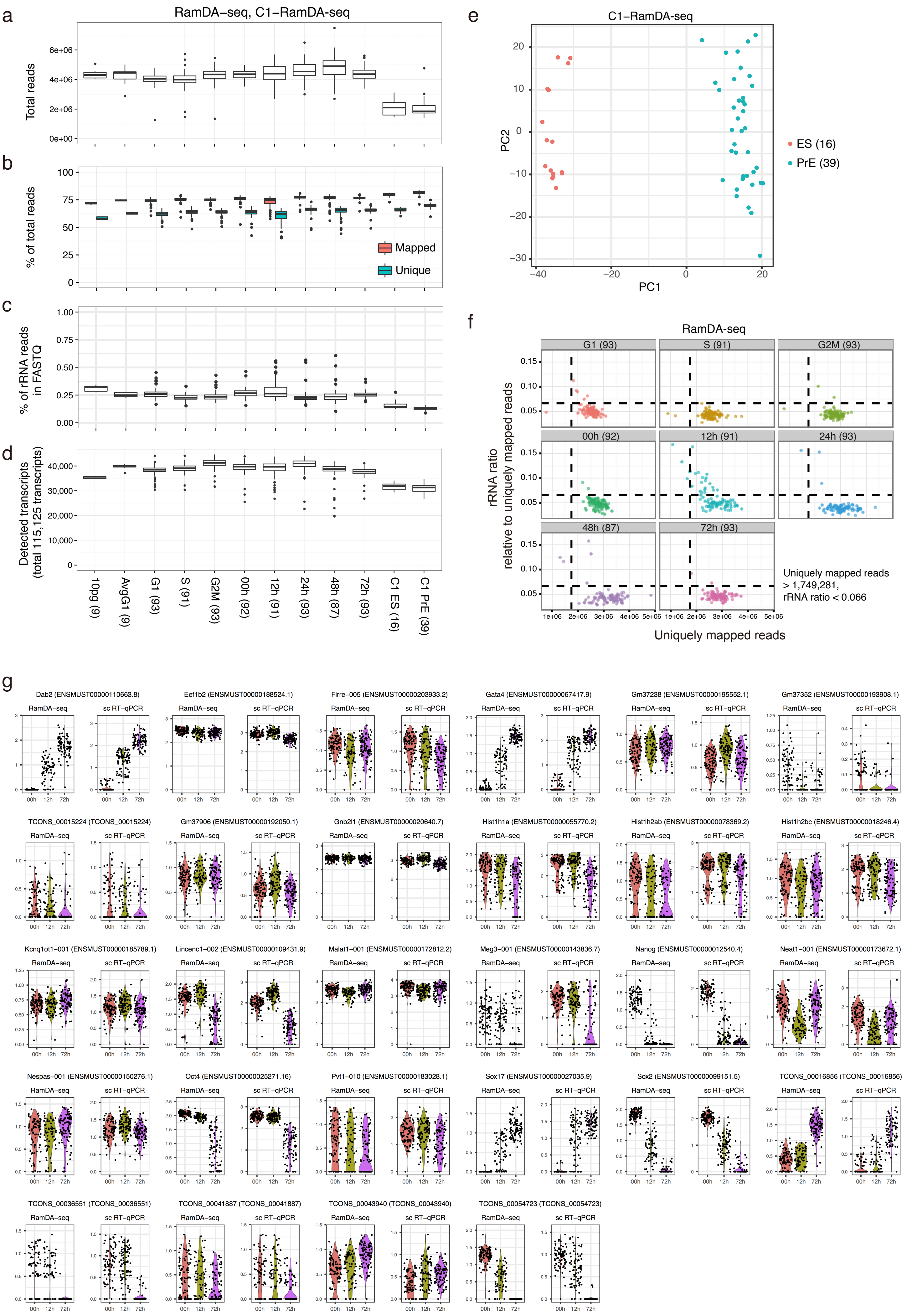

a
RamDA−seq, C1−RamDA-seq
Total reads
b
% of total reads
Mapped
Unique
c
% of rRNA reads in FASTQ
d
Detected transcripts (total 115,125 transcripts)
10pg (9)
AvgG1 (9)
G1 (93)
S (91)
G2M (93)
00h (92)
12h (91)
24h (93)
48h (87)
72h (93)
C1 ES (16)
C1 PrE (39)
e
C1−RamDA-seq
PC1
PC2
ES (16)
PrE (39)
f
RamDA-seq
rRNA ratio relative to uniquely mapped reads
Uniquely mapped reads
Uniquely mapped reads > 1,749,281, rRNA ratio < 0.066
g
Dab2 (ENSMUST00000110663.8)
Eef1b2 (ENSMUST00000188524.1)
Firre−005 (ENSMUST00000203933.2)
Gata4 (ENSMUST00000067417.9)
Gm37238 (ENSMUST00000195552.1)
Gm37352 (ENSMUST00000193908.1)
TCONS_00015224 (TCONS_00015224)
Gm37906 (ENSMUST00000192050.1)
Gnb2l1 (ENSMUST00000020640.7)
Hist1h1a (ENSMUST00000055770.2)
Hist1h2ab (ENSMUST00000078369.2)
Hist1h2bc (ENSMUST00000018246.4)
Kcnq1ot1−001 (ENSMUST00000185789.1)
Lincenc1−002 (ENSMUST00000109431.9)
Malat1−001 (ENSMUST00000172812.2)
Meg3−001 (ENSMUST00000143836.7)
Nanog (ENSMUST00000012540.4)
Neat1−001 (ENSMUST00000173672.1)
Nespas−001 (ENSMUST00000150276.1)
Oct4 (ENSMUST00000025271.16)
Pvt1−010 (ENSMUST00000183028.1)
Sox17 (ENSMUST00000027035.9)
Sox2 (ENSMUST00000099151.5)
TCONS_00016856 (TCONS_00016856)
TCONS_00036551 (TCONS_00036551)
TCONS_00041887 (TCONS_00041887)
TCONS_00043940 (TCONS_00043940)
TCONS_00054723 (TCONS_00054723)
RamDA−seq
sc RT−qPCR

Supplementary Figure 15. Quality assessment of RamDA-seq and C1-RamDA-seq samples

(**a**) Number of reads after adapter trimming for RamDA-seq and C1-RamDA-seq data. AvgG1: 'averaged' single-cell lysate samples from 180 pooled mESCs in the G1 phase. (**b**) Ratio of mapped and uniquely mapped reads relative to total reads. (**c**) The percentage of rRNA reads in FASTQ files. (**d**) Number of detected transcripts (TPM ≥ 0.1) determined by sailfish. (**e**) PCA analysis of C1-RamDA-seq data with ES and PrE cells. Note that ES and PrE cells were clearly separated, which indicated that C1-RamDA-seq could be successfully applied to living single cells. (**a-d**) The center line, lower and upper bounds of each box represent the median, first and third quartiles, respectively. The lower (upper) whisker extends to smallest (largest) values no further than 1.5 * IQR from the first (third) quartile. (**a-e**) The numbers in parentheses represent the number of cells. (**f**) Definition of 'outlier' cells. The *x*-axis represents the number of uniquely mapped reads. The *y*-axis represents the fraction of reads that were mapped to the rRNA annotations relative to the uniquely mapped reads. The vertical dashed lines (1,749,271; Q1 - 1.5 * IQR) represent the maximal value used for filtering cells. The horizontal dashed lines (0.066; Q3 + 1.5 * IQR) represent the minimal value used for filtering cells. ratioMax = dtnew2[, 1.5*(quantile(ratio, probs = 0.75)-quantile(ratio, probs = 0.25)) + quantile(ratio, probs = 0.75)]. The numbers in parentheses represent the number of cells. (**g**) Validation of the expression quantification measured by RamDA-seq using single-cell preamplification RT-qPCR (scRT-qPCR). Each point represents one cell in samples from 0, 12, and 72 h. The *y*-axes represent the log10(TPM+1) for RamDA-seq and the log10 (copy number + 1) for scRT-qPCR.

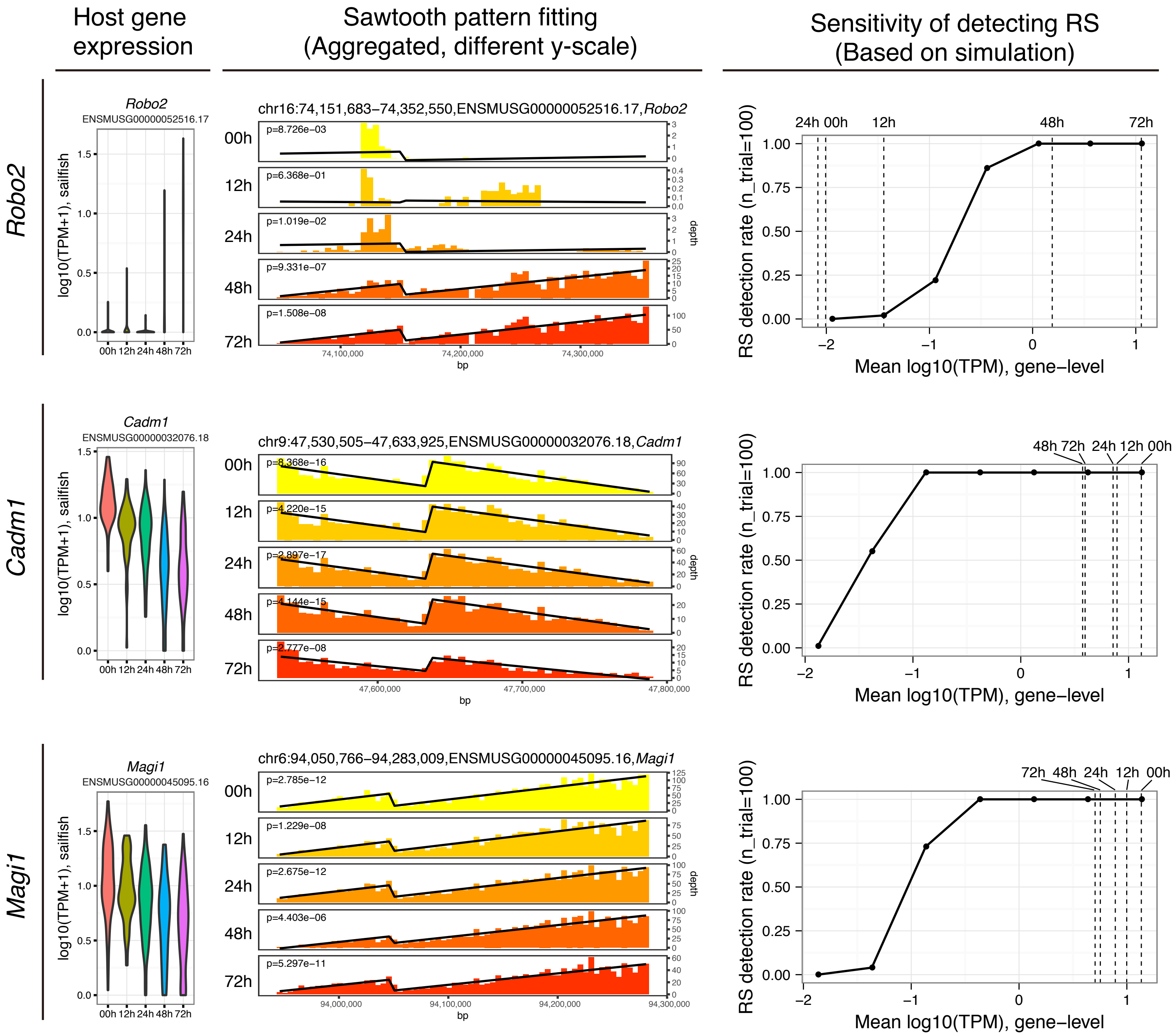

Host gene expression
Sawtooth pattern fitting (Aggregated, different y-scale)
Sensitivity of detecting RS (Based on simulation)
Robo2
ENSMUSG00000052516.17
log10(TPM+1), sailfish
00h 12h 24h 48h 72h
chr16:74,151,683–74,352,550,ENSMUSG00000052516.17,Robo2
p=8.726e−03
p=6.368e−01
p=1.019e−02
p=9.331e−07
p=1.508e−08
depth
bp
RS detection rate (n_trial=100)
Mean log10(TPM), gene−level
Cadm1
ENSMUSG00000032076.18
chr9:47,530,505–47,633,925,ENSMUSG00000032076.18,Cadm1
p=8.368e−16
p=4.220e−15
p=2.897e−17
p=4.144e−15
p=2.777e−08
Magi1
ENSMUSG00000045095.16
chr6:94,050,766–94,283,009,ENSMUSG00000045095.16,Magi1
p=2.785e−12
p=1.229e−08
p=2.675e−12
p=4.403e−06
p=5.297e−11

Supplementary Figure 16. Analyses of recursive splicing

Analyses of recursive splicing of *Robo2*, *Cadm1*, and *Magi1*. (Left) Violin plots showing the expression levels of host genes for each cell. (Middle) The summed normalized read coverage of RamDA-seq for each time point in the 5-kbp bin (bars) and fitted linear regression models (black lines). The p-values of F-tests are indicated. Note that the y-axes vary for different time points. (Right) Simulation-based estimation of sensitivity for detecting RS. The summed normalized read coverage of RamDA-seq for 72 h (*Robo2*) or 00 h (*Cadm1* and *Magi1*) were subsampled 100 times for different fractions. For each subsampling fraction, the subsampled read coverage data were used to detect RS and calculate the RS detection rate as the fraction of RS. The x-axis represents the subsampling fraction converted to the gene-level expression levels of host genes. Dashed lines indicate normalized coverage for time points.

a

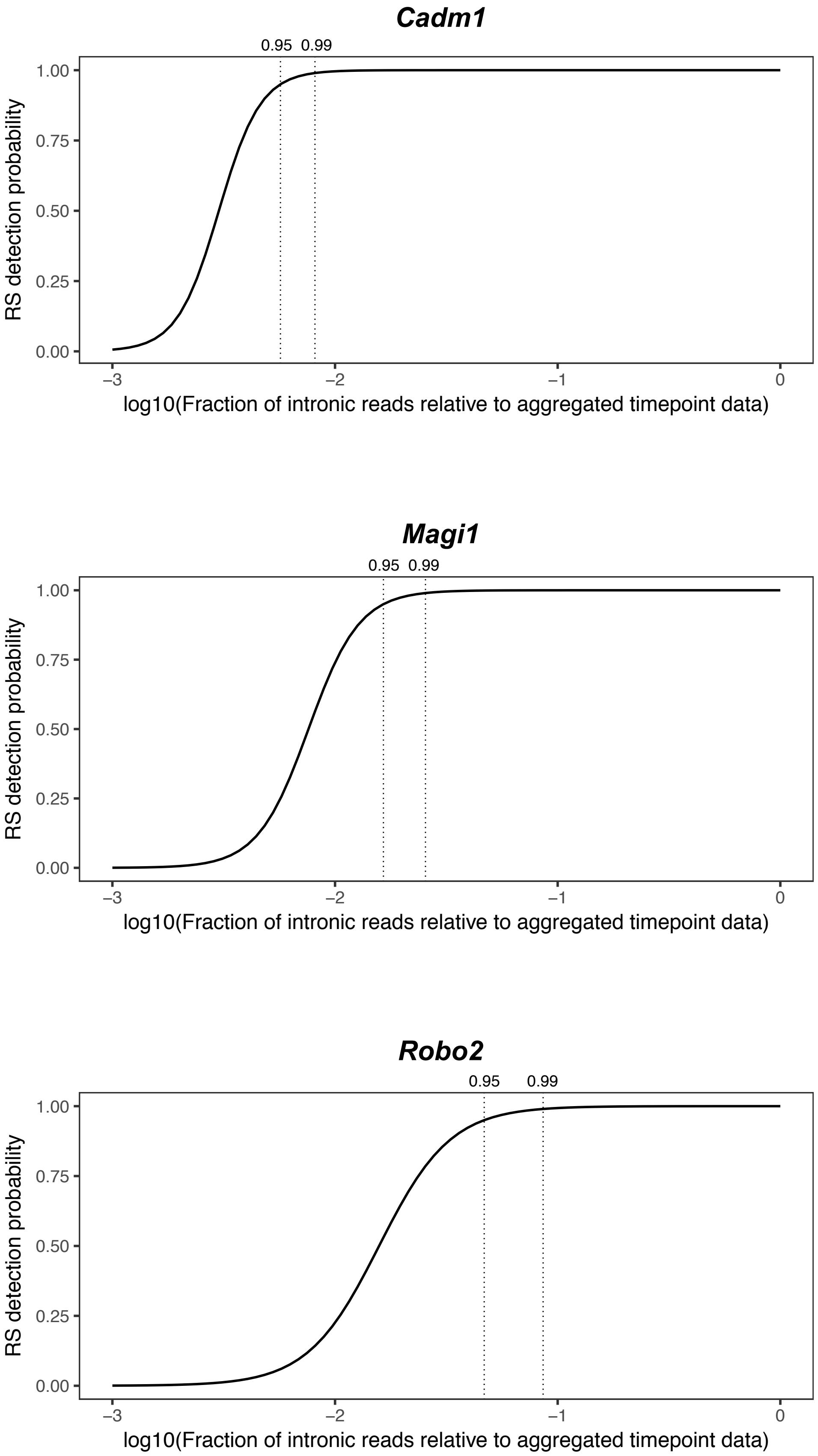

Cadm1
0.95
0.99
RS detection probability
1.00
0.75
0.50
0.25
0.00
−3
−2
−1
0
log10(Fraction of intronic reads relative to aggregated timepoint data)
Magi1
0.95
0.99
RS detection probability
1.00
0.75
0.50
0.25
0.00
−3
−2
−1
0
log10(Fraction of intronic reads relative to aggregated timepoint data)
Robo2
0.95
0.99
RS detection probability
1.00
0.75
0.50
0.25
0.00
−3
−2
−1
0
log10(Fraction of intronic reads relative to aggregated timepoint data)

b

Probability = 0.95

chr9:47530505–47633925,ENSMUSG00000032076.18,Cadm1

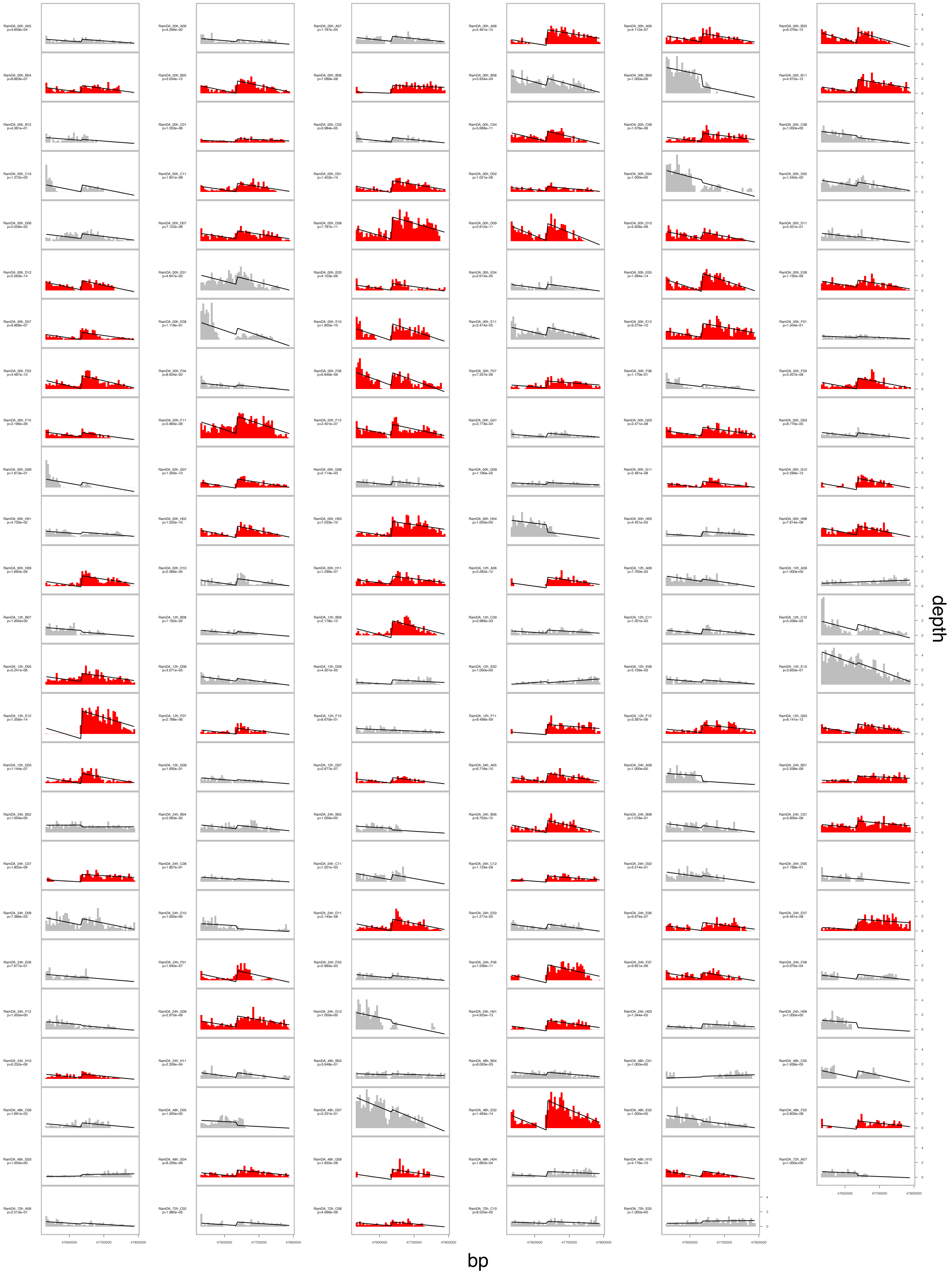

Probability = 0.95

c

chr6:94050766–94283009,ENSMUSG00000045095.16,Magi1

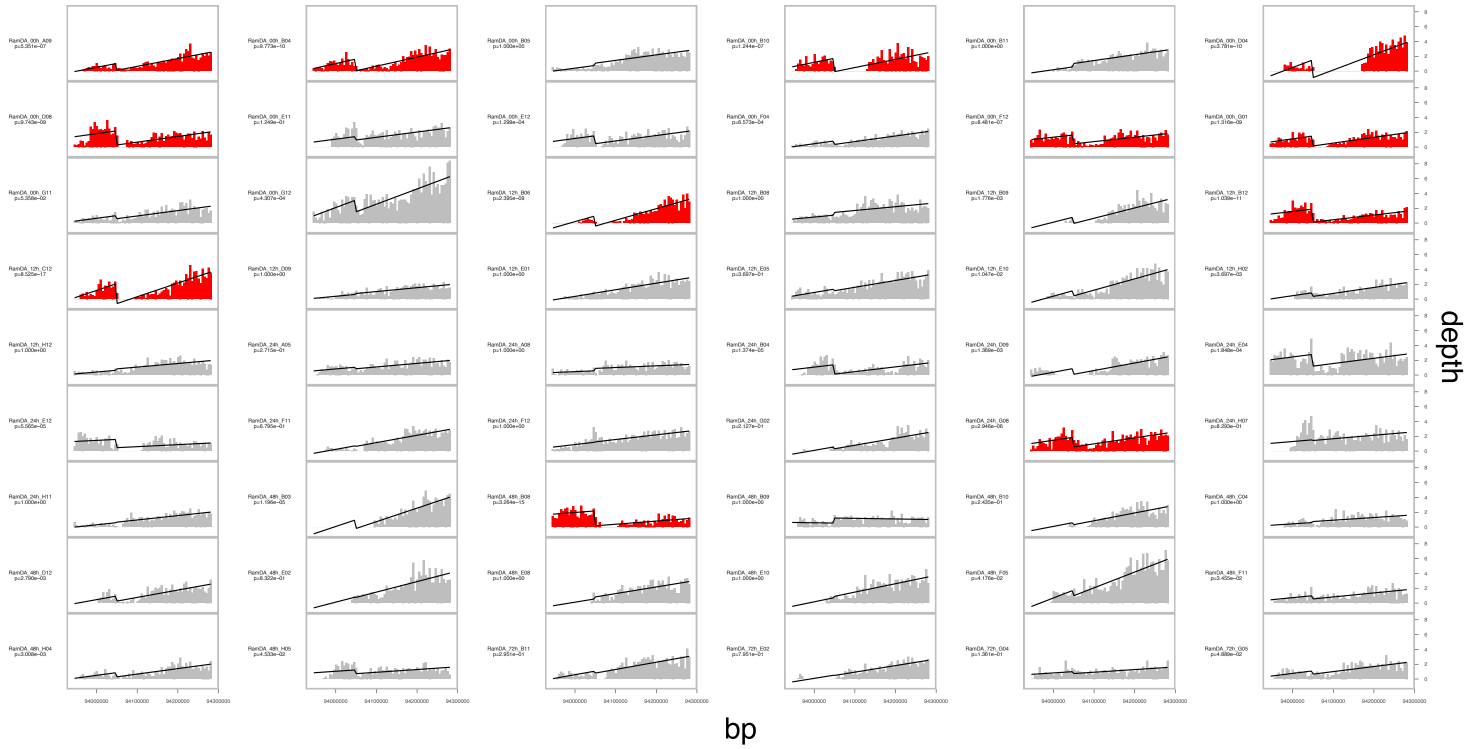

d

chr16:74151683–74352550,ENSMUSG00000052516.17,Robo2

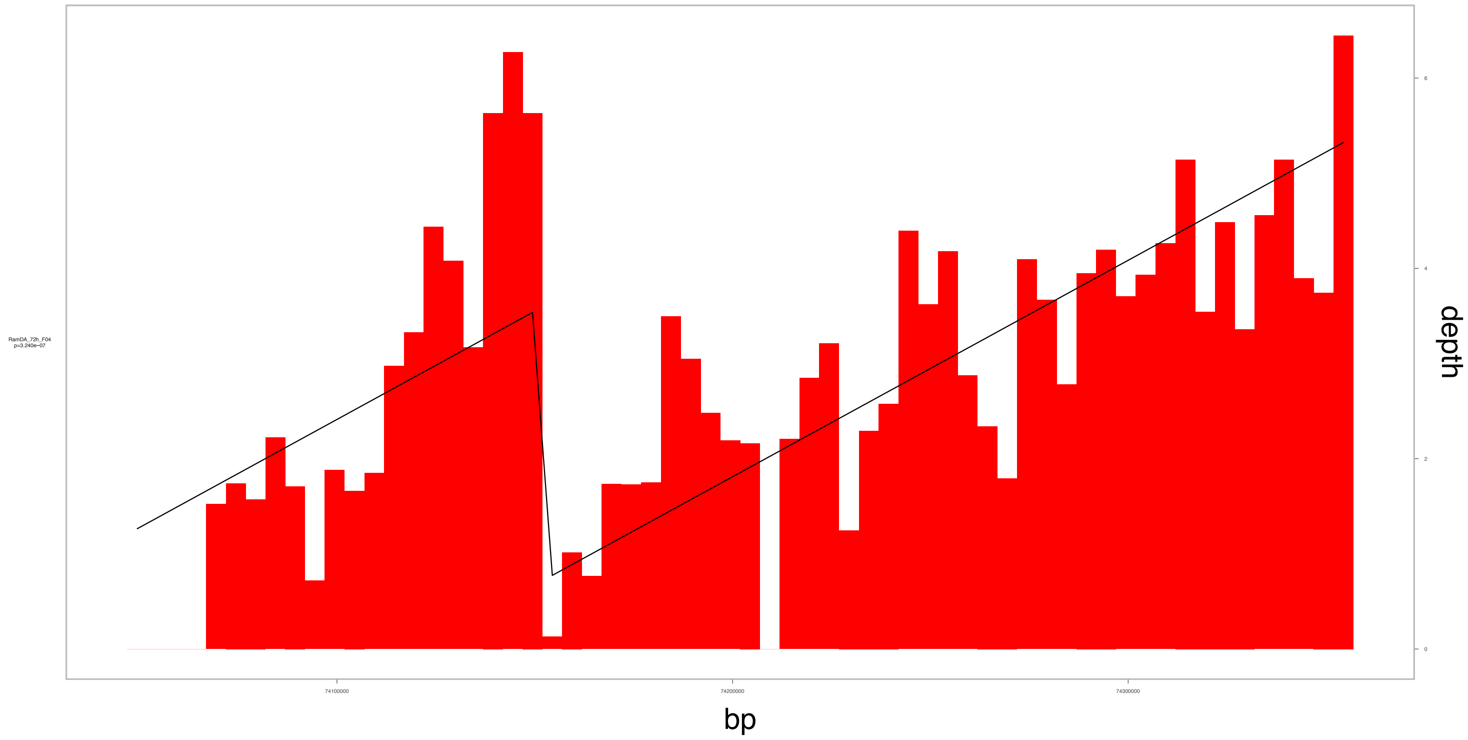

Probability = 0.99

e

chr9:47530505–47633925,ENSMUSG00000032076.18,Cadm1

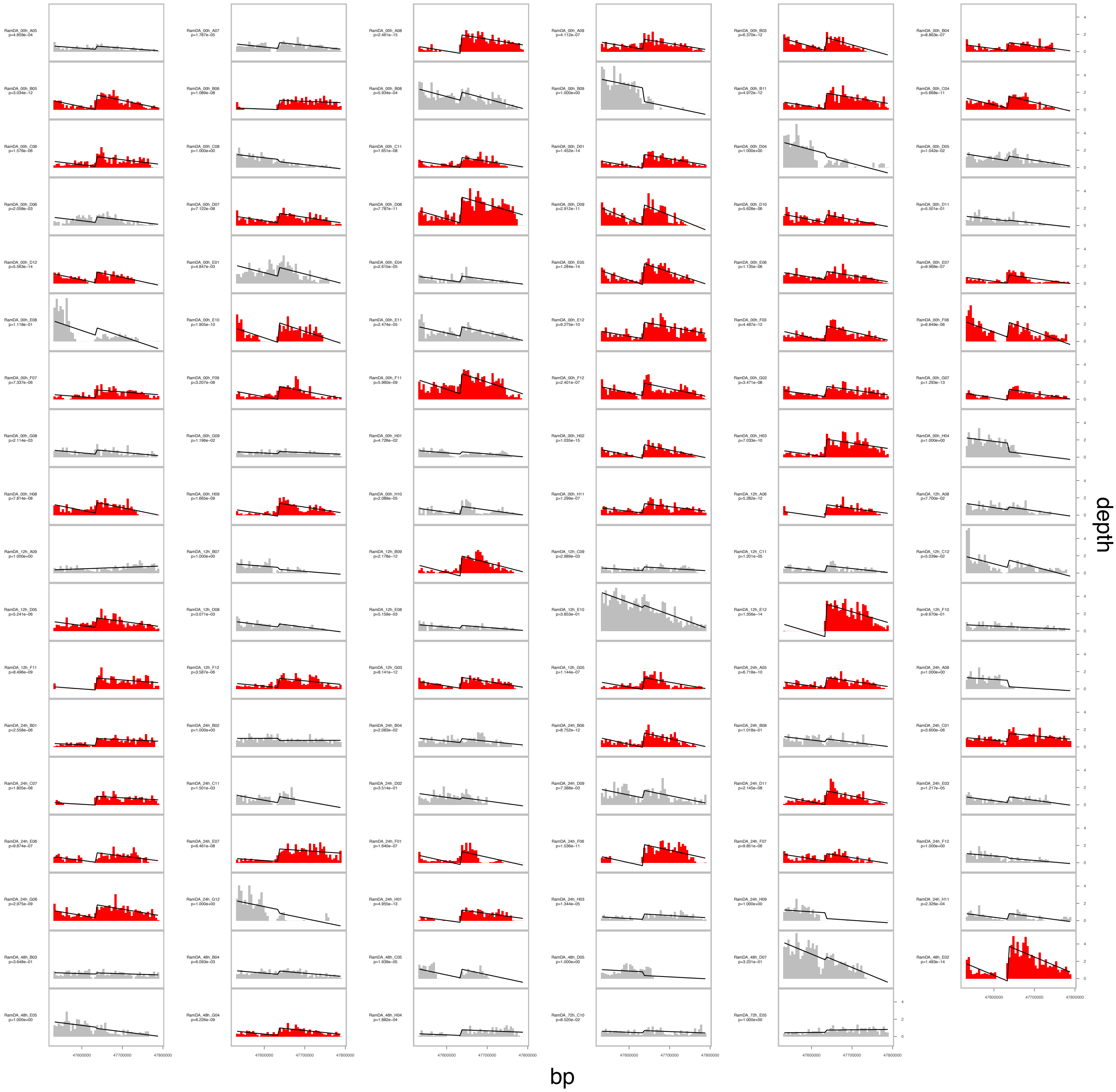

f

chr6:94050766–94283009,ENSMUSG00000045095.16,Magi1

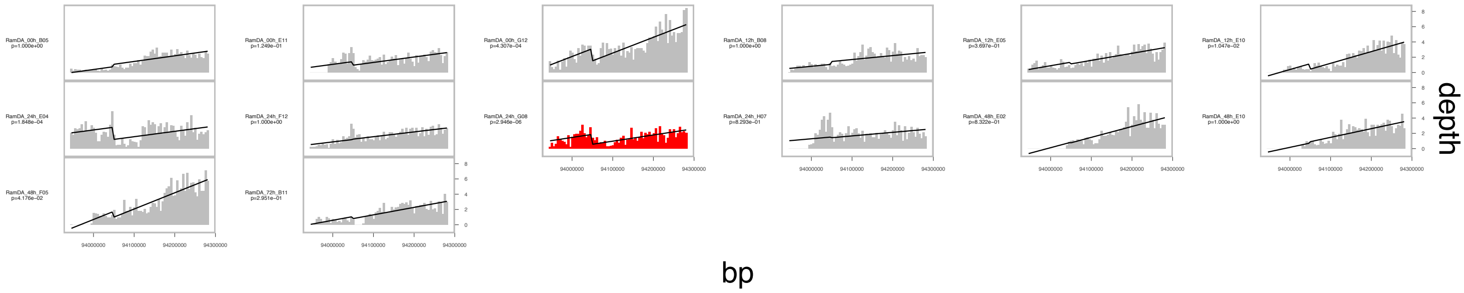

Supplementary Figure 17. Detection of recursive splicing in individual single cells (**a**) RS detection probability given the fraction of intronic reads relative to aggregated timepoint data (00 h for *Cadm1* and *Magi1*, 72 h for *Robo2*). The dashed lines in each panel represent the fraction corresponding to the probability of 0.95 and 0.99. (**b-f**) Read coverage of introns. Red and gray bars indicate significance ($p < 1e-5$) or no significance, respectively. Only cells with enough intronic reads (RS detection probability > 0.95 (**b-d**) or 0.99 (**e-f**)) and reads in introns both upstream and downstream of the RS site were considered.

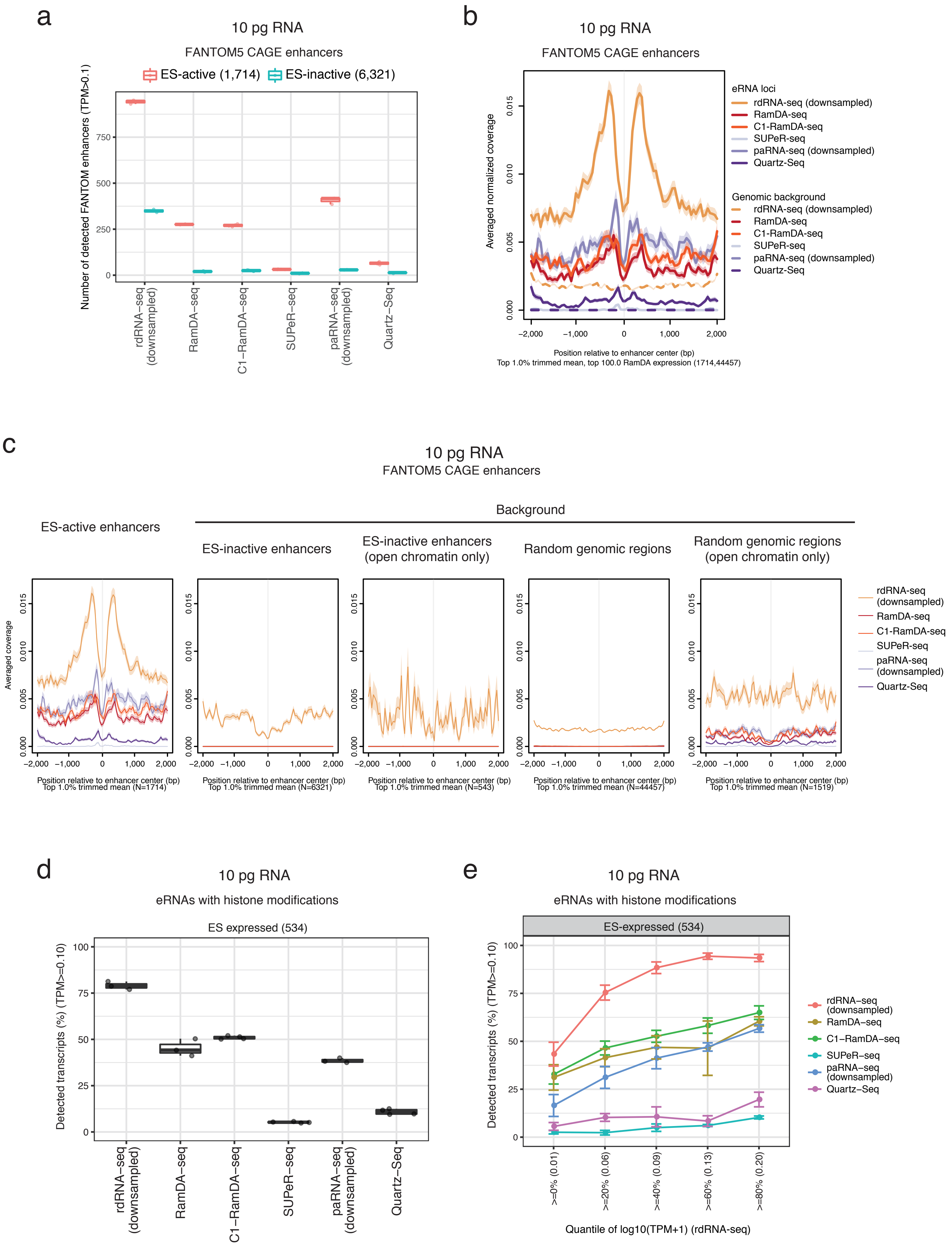
a
10 pg RNA
FANTOM5 CAGE enhancers
ES-active (1,714)
ES-inactive (6,321)
Number of detected FANTOM enhancers (TPM>0.1)
0
250
500
750
rdRNA–seq (downsampled)
RamDA–seq
C1–RamDA–seq
SUPeR–seq
paRNA–seq (downsampled)
Quartz–Seq
b
10 pg RNA
FANTOM5 CAGE enhancers
Averaged normalized coverage
eRNA loci
rdRNA-seq (downsampled)
RamDA-seq
C1-RamDA-seq
SUPeR-seq
paRNA-seq (downsampled)
Quartz-Seq
Genomic background
rdRNA-seq (downsampled)
RamDA-seq
C1-RamDA-seq
SUPeR-seq
paRNA-seq (downsampled)
Quartz-Seq
Position relative to enhancer center (bp)
Top 1.0% trimmed mean, top 100.0 RamDA expression (1714,44457)
c
10 pg RNA
FANTOM5 CAGE enhancers
ES-active enhancers
Background
ES-inactive enhancers
ES-inactive enhancers (open chromatin only)
Random genomic regions
Random genomic regions (open chromatin only)
Averaged coverage
Position relative to enhancer center (bp)
Top 1.0% trimmed mean (N=1714)
Top 1.0% trimmed mean (N=6321)
Top 1.0% trimmed mean (N=543)
Top 1.0% trimmed mean (N=44457)
Top 1.0% trimmed mean (N=1519)
rdRNA-seq (downsampled)
RamDA-seq
C1-RamDA-seq
SUPeR-seq
paRNA-seq (downsampled)
Quartz-Seq
d
10 pg RNA
eRNAs with histone modifications
ES expressed (534)
Detected transcripts (%) (TPM>=0.10)
rdRNA–seq (downsampled)
RamDA–seq
C1–RamDA–seq
SUPeR–seq
paRNA–seq (downsampled)
Quartz–Seq
e
10 pg RNA
eRNAs with histone modifications
ES-expressed (534)
Detected transcripts (%) (TPM>=0.10)
>=0% (0.01)
>=20% (0.06)
>=40% (0.09)
>=60% (0.13)
>=80% (0.20)
Quantile of log10(TPM+1) (rdRNA-seq)
rdRNA–seq (downsampled)
RamDA–seq
C1–RamDA–seq
SUPeR–seq
paRNA–seq (downsampled)
Quartz–Seq

Supplementary Figure 18. Enhancer RNA detection using scRNA-seq data from 10 pg RNA

(**a**) Number of detected FANTOM5 enhancers by scRNA-seq methods for ES-active (red) and ES-inactive (blue) subsets. The ES-active and ES-inactive sets were defined using CAGE data for mESCs from the FANTOM5 project. The numbers in parentheses represent the number of enhancers. (**b**) Aggregation plot for the read coverage around the ES-active enhancers (solid lines) and random genomic regions (dashed lines). Normalized read coverage was averaged across the cells and normalized such that the sum of each enhancer was 1 and further averaged across enhancers after trimming the top 1% of enhancers for each 50-bp bin. The shaded areas represent standard deviations. The numbers in parentheses represent the number of enhancers or background regions. (**c**) Aggregation plot for the read coverage around the ES-active enhancers (left) and different background regions. The numbers in parentheses represent the number of enhancers or background regions. (**d**) The detection rates of non-poly(A) (loose criterion) eRNAs with enhancer-like histone modifications expressed in ES cells. The number in parentheses represents the number of eRNAs. For the boxplots in (**a**) and (**d**), the center line, lower and upper bounds of each box represent the median, first and third quartiles, respectively. The lower (upper) whisker extends to smallest (largest) values no further than 1.5 * IQR from the first (third) quartile. (**e**) Detection rates of non-poly(A) (loose criterion) eRNAs as described in (**d**) as a function of rdRNA-seq expression levels. The points and error bars represent means and standard deviations, respectively. Each line represents a scRNA-seq method. The number in parentheses represents the number of eRNAs.

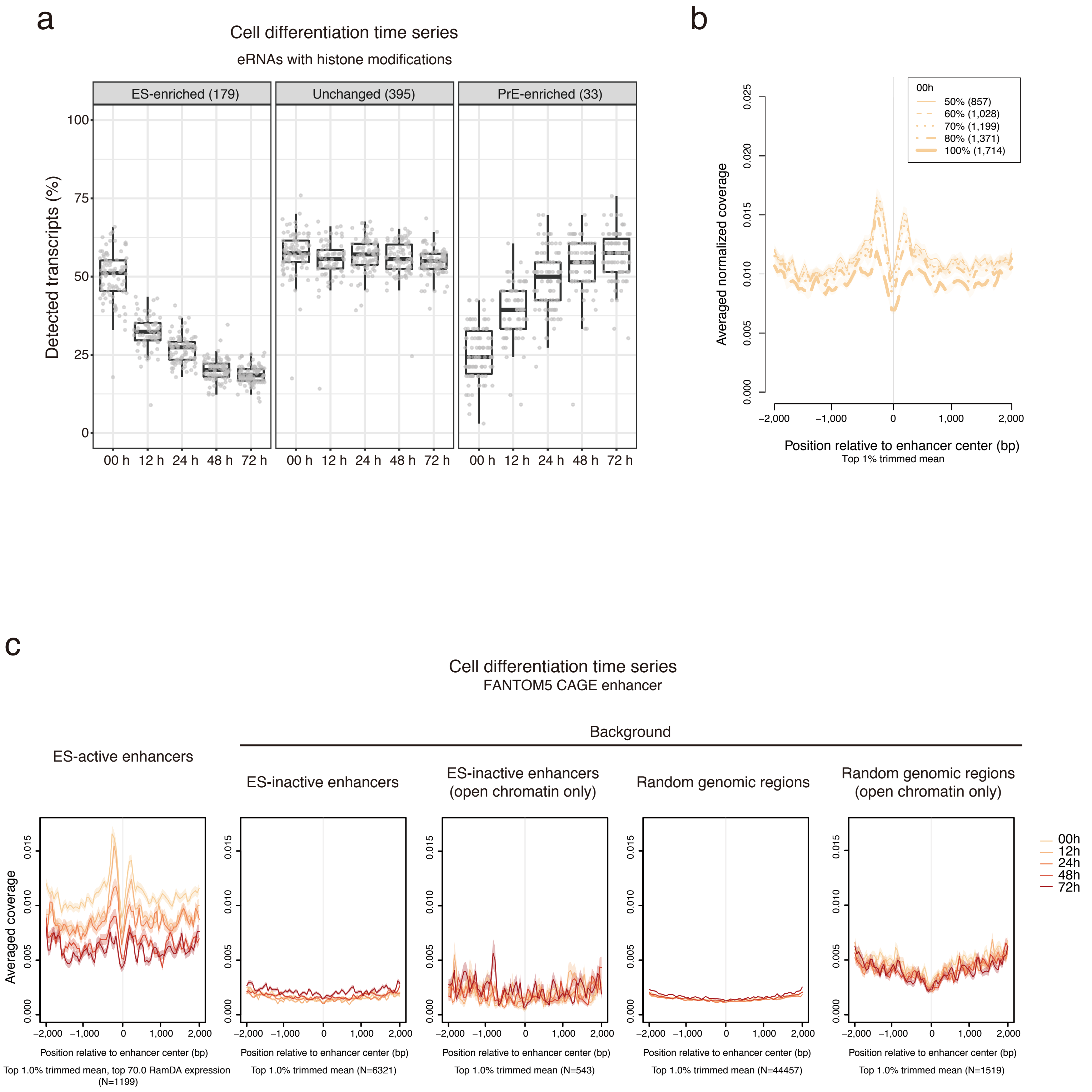
a
Cell differentiation time series
eRNAs with histone modifications
ES-enriched (179)
Unchanged (395)
PrE-enriched (33)
Detected transcripts (%)
100
75
50
25
0
00 h
12 h
24 h
48 h
72 h
b
00h
50% (857)
60% (1,028)
70% (1,199)
80% (1,371)
100% (1,714)
Averaged normalized coverage
0.000
0.005
0.010
0.015
0.020
0.025
−2,000
−1,000
0
1,000
2,000
Position relative to enhancer center (bp)
Top 1% trimmed mean
c
Cell differentiation time series
FANTOM5 CAGE enhancer
Background
ES-active enhancers
ES-inactive enhancers
ES-inactive enhancers (open chromatin only)
Random genomic regions
Random genomic regions (open chromatin only)
Averaged coverage
0.000
0.005
0.010
0.015
00h
12h
24h
48h
72h
Position relative to enhancer center (bp)
Top 1.0% trimmed mean, top 70.0 RamDA expression (N=1199)
Top 1.0% trimmed mean (N=6321)
Top 1.0% trimmed mean (N=543)
Top 1.0% trimmed mean (N=44457)
Top 1.0% trimmed mean (N=1519)

Supplementary Figure 19. Enhancer RNA detection using time-series RamDA-seq data

(**a**) The number of detected non-poly(A) eRNAs for ES-enriched (left), unchanged (middle), and PrE-enriched (right) subsets. Each point represents the ratio of detected eRNAs per cell. The numbers in parentheses represent the number of eRNAs. The center line, lower and upper bounds of each box represent the median, first and third quartiles, respectively. The lower (upper) whisker extends to smallest (largest) values no further than 1.5 * IQR from the first (third) quartile. (**b**) Averaged normalized coverage of RamDA-seq samples at 00 h for the subset of ES-active enhancers. When the narrower threshold was applied (top 100, 80, 70, 60, and 50% of expression levels measured by RamDA-seq), the bimodal peaks became clearer. The numbers in parentheses represent the number of enhancers. (**c**) Aggregation plot of the read coverage of RamDA-seq data around the ES-active enhancers (left) and different background regions. Normalized read coverage was averaged across the cells and normalized such that the sum of each enhancer was 1 and further averaged across enhancers after trimming the top 1% of enhancers for each 50-bp bin. Only the top 70% of highly expressed enhancers (quantified within regions 75 to 275 bp away from the center of enhancers) was included. The shaded areas represent standard deviations. The numbers in parentheses represent the number of enhancers or background regions.

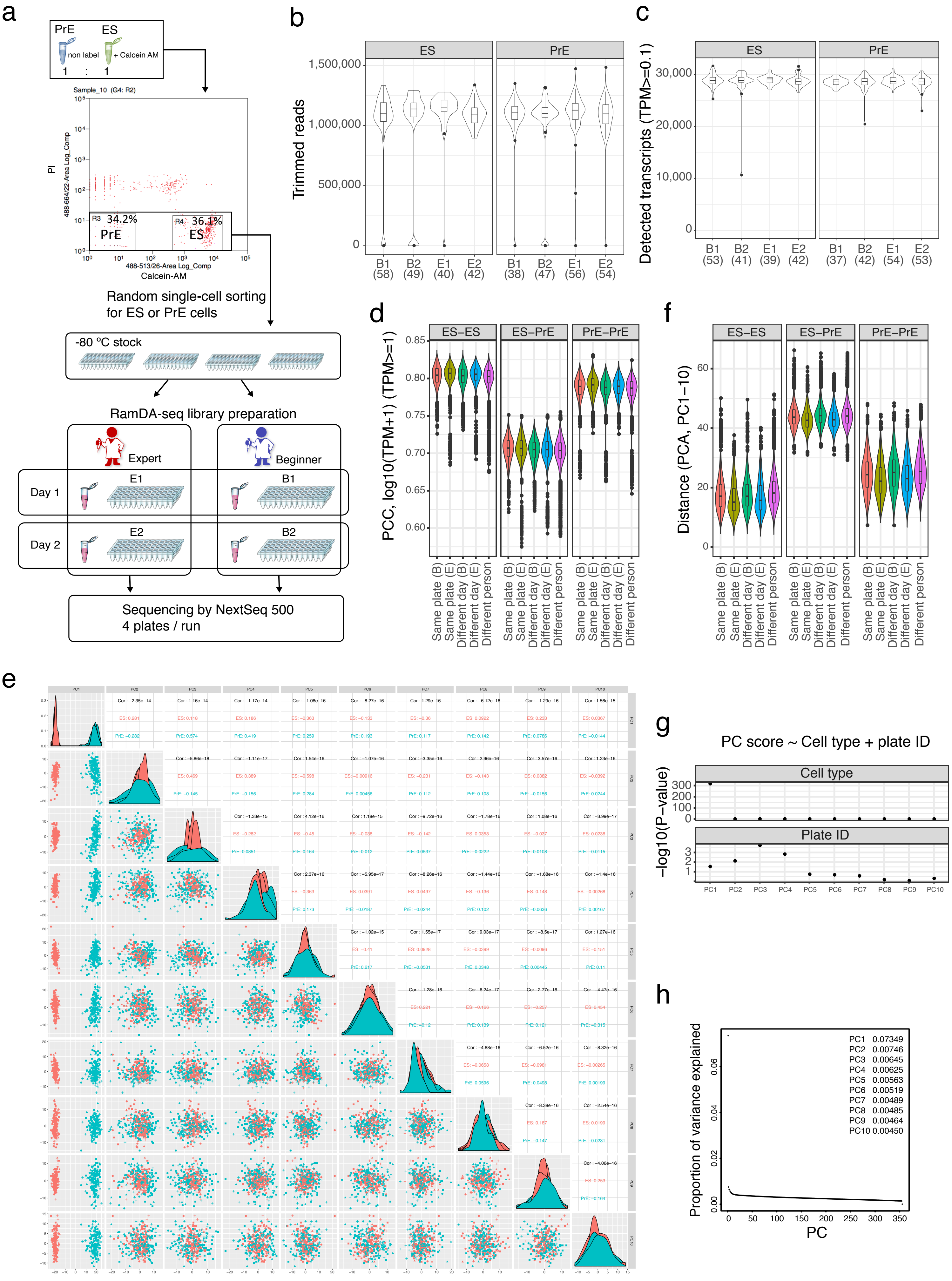
a
PrE
ES
non label
+ Calcein AM
1 : 1
Sample_10 (G4: R2)
PI
488-664/22-Area Log_Comp
R3 34.2%
PrE
R4 36.1%
ES
488-513/26-Area Log_Comp
Calcein-AM
Random single-cell sorting
for ES or PrE cells
-80 ºC stock
RamDA-seq library preparation
Expert
Beginner
Day 1
Day 2
E1
B1
E2
B2
Sequencing by NextSeq 500
4 plates / run
b
Trimmed reads
ES
PrE
1,500,000
1,000,000
500,000
0
B1 (58)
B2 (49)
E1 (40)
E2 (42)
B1 (38)
B2 (47)
E1 (56)
E2 (54)
c
Detected transcripts (TPM>=0.1)
ES
PrE
30,000
20,000
10,000
0
B1 (53)
B2 (41)
E1 (39)
E2 (42)
B1 (37)
B2 (42)
E1 (54)
E2 (53)
d
PCC, log10(TPM+1) (TPM>=1)
ES–ES
ES–PrE
PrE–PrE
0.85
0.80
0.75
0.70
0.65
0.60
Same plate (B)
Same plate (E)
Different day (B)
Different day (E)
Different person
f
Distance (PCA, PC1−10)
ES–ES
ES–PrE
PrE–PrE
60
40
20
0
Same plate (B)
Same plate (E)
Different day (B)
Different day (E)
Different person
e
PC1
PC2
PC3
PC4
PC5
PC6
PC7
PC8
PC9
PC10
g
PC score ~ Cell type + plate ID
−log10(P−value)
Cell type
Plate ID
PC1
PC2
PC3
PC4
PC5
PC6
PC7
PC8
PC9
PC10
h
Proportion of variance explained
PC
PC1 0.07349
PC2 0.00746
PC3 0.00645
PC4 0.00625
PC5 0.00563
PC6 0.00519
PC7 0.00489
PC8 0.00485
PC9 0.00464
PC10 0.00450

Supplementary Figure 20. Analyses of batch effect

Plate-to-plate variability was investigated. Although variabilities between plates were larger than variabilities within plates, plate-to-plate variability was much smaller than variability between cell types, and the proportion of variance explained in PCA was 0.6%. (**a**) Experimental design to study plate-to-plate variability. ES and PrE cells were randomly sorted into four 96-well plates. Each of two experimenters (an expert and a beginner) performed RamDA-seq with one plate on two separate days (Day 1 and 2), yielding 4 plates of RamDA-seq data (E1, E2, B1, and B2). The reagent mixture was independently prepared by each experimenter every experiment day. (**b**) The number of reads after adapter trimming. Outlier cells were identified as cells with the number of trimmed reads lower than Q1 - 1.5*(IQR). The numbers in parentheses represent the number of cells. (**c**) The number of detected transcripts (TPM ≥ 0.1). Outlier cells were further identified as cells with the number of detected transcripts lower than Q1 - 1.5*(IQR). The numbers in parentheses represent the number of cells. (**d**) Pearson correlation coefficients of various pairs of cells (62,835 pairs). Three combinations of cell types (ES-ES, ES-PrE, and PrE-PrE) are indicated. Same batch: Pairs of cells within the same plates. Different day: Pairs of cells from different plates from the same experimenters. Different person: Pairs of cells from different plates from different experiments. B: Beginner, E: Expert. Transcripts with TPM ≥ 1 in both cells of each pair were used. (**e**) Pair plots of PCA of cells from 4 plates. Red and turquoise points represent ES and PrE cells, respectively. Different shapes indicate different plates. (**f**) The Euclidian distances between two cells from different pairs (62,835 pairs) as (d). The Euclidian distances were calculated based on the vectors of principal component scores of PC1-10. (**g**) P-values of F-tests for two-way analysis of variance (ANOVA). An additive model was assumed in which cell types and plate IDs (B1, B2, E1, and E2) were categorical independent variables, and the principal component score of each PC axis was a continuous dependent variable. (**h**) Proportion of variance explained for PCA.

Supplementary References

1. Armour, C. D. *et al.* Digital transcriptome profiling using selective hexamer priming for cDNA synthesis. *Nat. Methods* 6, 647–649 (2009).
2. Ozsolak, F. *et al.* Digital transcriptome profiling from attomole-level RNA samples. *Genome Res.* 20, 519–525 (2010).
3. Sasagawa, Y. *et al.* Quartz-Seq: a highly reproducible and sensitive single-cell RNA sequencing method, reveals non-genetic gene-expression heterogeneity. *Genome Biol.* 14, R31 (2013).
4. Fan, X. *et al.* Single-cell RNA-seq transcriptome analysis of linear and circular RNAs in mouse preimplantation embryos. *Genome Biol.* 16, 148 (2015).
5. Ramsköld, D. *et al.* Full-length mRNA-Seq from single-cell levels of RNA and individual circulating tumor cells. *Nat. Biotechnol.* 30, 777–782 (2012).
6. Picelli, S. *et al.* Smart-seq2 for sensitive full-length transcriptome profiling in single cells. *Nat. Methods* 10, 1096–1098 (2013).
7. Islam, S. *et al.* Characterization of the single-cell transcriptional landscape by highly multiplex RNA-seq. *Genome Res.* 21, 1160–1167 (2011).
8. Islam, S. *et al.* Quantitative single-cell RNA-seq with unique molecular identifiers. *Nat. Methods* 11, 163–166 (2013).
9. Zhang, Y. *et al.* Histone h1 depletion impairs embryonic stem cell differentiation. *PLoS Genet.* 8, e1002691 (2012).
10. Marzluff, W. F. & Duronio, R. J. Histone mRNA expression: multiple levels of cell cycle regulation and important developmental consequences. *Curr. Opin. Cell Biol.* 14, 692-699 (2002).
11. Moore, R., Tao, W., Smith, E. R. & Xu, X.-X. The primitive endoderm segregates from the epiblast in β1 integrin-deficient early mouse embryos. *Mol. Cell. Biol.* 34, 560–572 (2014).
12. Niakan, K. K. *et al.* Sox17 promotes differentiation in mouse embryonic stem cells by directly regulating extraembryonic gene expression and indirectly antagonizing self-renewal. *Genes Dev.* 24, 312–326 (2010).
13. Andersson, R. *et al.* An atlas of active enhancers across human cell types and tissues. *Nature* 507, 455–461 (2014).
14. Djebali, S. *et al.* Landscape of transcription in human cells. *Nature* 489, 101–108 (2012).
