# Supplementary material for: Single-cell full-length total RNA sequencing uncovers dynamics of recursive splicing and enhancer RNAs
Source: Nat Commun. 2018 Feb 12;9:619. doi: 10.1038/s41467-018-02866-0 (PMC5809388; doi:10.1038/s41467-018-02866-0)
Supplement: Supplementary file 3 — Description of Additional Supplementary Files [file 41467_2018_2866_MOESM3_ESM.pdf]

Description of Additional Supplementary Files

File Name: Supplementary Data 1
Description: Functional term enrichment analysis using MetaScape.

File Name: Supplementary Data 2
Description: List of qRT-PCR primers.

File Name: Supplementary Data 3
Description: List of not-so-random primers.

File Name: Supplementary Data 4
Description: : List of non-poly(A) RNAs.

File Name: Supplementary Data 5
Description: List of poly(A) RNAs.

File Name: Supplementary Data 6
Description: Mouse ribosomal RNA sequences.
